# Making sense of the linear genome, gene function and TADs

**DOI:** 10.1186/s13072-022-00436-9

**Published:** 2022-01-29

**Authors:** Helen S. Long, Simon Greenaway, George Powell, Ann-Marie Mallon, Cecilia M. Lindgren, Michelle M. Simon

**Affiliations:** 1 Nuffield Department of Medicine, University of Oxford, Oxford, UK; 2 Mammalian Genetics Unit, Harwell Institute, Didcot, UK; 3 Wellcome Centre for Human Genetics, University of Oxford, Oxford, UK; 4 Big Data Institute, Li Ka Shing Centre for Health Information and Discovery, University of Oxford, Oxford, UK; 5 Medical and Population Genetics Program, Broad Institute of MIT and Harvard, Cambridge, MA USA

**Keywords:** TADs, Topologically associating domains, Hi-C, Chromatin structure, Gene regulation, Gene function, Gene expression

## Abstract

**Background:**

Topologically associating domains (TADs) are thought to act as functional units in the genome. TADs co-localise genes and their regulatory elements as well as forming the unit of genome switching between active and inactive compartments. This has led to the speculation that genes which are required for similar processes may fall within the same TADs, allowing them to share regulatory programs and efficiently switch between chromatin compartments. However, evidence to link genes within TADs to the same regulatory program is limited.

**Results:**

We investigated the functional similarity of genes which fall within the same TAD. To do this we developed a TAD randomisation algorithm to generate sets of “random TADs” to act as null distributions. We found that while pairs of paralogous genes are enriched in TADs overall, they are largely depleted in TADs with CCCTC-binding factor (CTCF) ChIP-seq peaks at both boundaries. By assessing gene constraint as a proxy for functional importance we found that genes which singly occupy a TAD have greater functional importance than genes which share a TAD, and these genes are enriched for developmental processes. We found little evidence that pairs of genes in CTCF bound TADs are more likely to be co-expressed or share functional annotations than can be explained by their linear proximity alone.

**Conclusions:**

These results suggest that algorithmically defined TADs consist of two functionally different groups, those which are bound by CTCF and those which are not. We detected no association between genes sharing the same CTCF TADs and increased co-expression or functional similarity, other than that explained by linear genome proximity. We do, however, find that functionally important genes are more likely to fall within a TAD on their own suggesting that TADs play an important role in the insulation of these genes.

**Supplementary Information:**

The online version contains supplementary material available at 10.1186/s13072-022-00436-9.

## Background

The organisation of the mammalian genome in three-dimensional space is non-random and hierarchically organised [1]. Using Hi-C [2] it was shown that chromosomal loci are clustered into two, mega base scale structures known as the A and B compartments [3]. The A compartment is enriched for active, euchromatin, whereas the B compartment is enriched for inactive, heterochromatin [3, 4]. The formation of chromatin compartments is hypothesised to be driven by phase separation [5]. By analysing chromatin contact maps at kilobase resolution Dixon et al. were able to identify a finer level of chromatin organisation known as Topologically Associating Domains (TADs). TADs are regions of the genome characterised by a high degree of self-interaction within the length of the TAD, and a low degree of interactions with regions outside of the TAD even if they are a similar distance away [6]. TAD boundaries are enriched for CCCTC-binding factor (CTCF) binding sites and are thought to be formed by active loop extrusion, during which DNA is extruded through cohesin (forming a loop) until the extrusion is stalled by boundary proteins at the TAD boundaries. Commonly the boundary protein is CTCF bound at a pair of convergently orientated CTCF binding sites at the TAD boundaries [5–9]. Comparisons of TADs between tissues suggested that they are largely tissue invariant [4, 6, 10]. It has also been proposed that a third category of chromatin organisation exists, which nests within TADs, these ‘sub-TADs’, are formed by the same mechanisms as TADs but have weaker insulation and are more likely to vary depending on the cell type [11]. However, it is currently unclear whether sub-TADs constitute functionally different structures to TADs [11].

It has been proposed that TADs constitute functional units in the genome, important for correct regulation of gene expression. TADs co-localise regulatory elements with their target genes, and are thought to promote co-regulation of the genes they contain, creating “gene regulatory domains” [12]. By inserting regulatory sensors along the length of the genome, Symmons et al. found evidence that the activity of enhancers is split into regulatory domains which highly correlate with TADs [13]. This provided experimental evidence that TADs potentially facilitate enhancers to carry out “non-specific” co-regulation of all genes in the TAD [12, 13]. Simultaneously, TADs are thought to insulate genes from aberrant regulation by regulatory elements outside the TAD (enhancer hijacking) [12]. Several examples of congenital disease have been linked to TAD boundary disruptions allowing enhancer hijacking demonstrating that at least in these cases, TAD boundaries are essential for proper gene regulation [14, 15]. TAD boundaries are also able to block the spread of transcription, and repressive chromatin [12]. It has been observed that the unit of compartment switching in the genome tends to be a single or series of TADs [16]. Adding to this picture, it has been suggested that genes within the same TAD have highly correlated expression patterns [17–20]. This has led to the speculation that genes which are required for specific processes may be contained within the same TAD to allow them to share regulatory programs and efficiently switch between the active and inactive compartments [21]. Studies have already indicated that some TADs may be enriched for lineage-specific genes [21, 22], but the global relationship between TADs and gene function is yet to be fully understood.

It has long been known that the linear order of genes in the genome is non-random with respect to gene function. Genes that are close together in linear space are more likely to have correlated expression patterns [23], and share pathways and protein–protein interactions (PPI) [24]. Genes within TADs are by definition also close together in the linear genome therefore the linear proximity between genes is an important confounding factor when studying the similarity of genes that share a TAD. It is also possible that the increased similarity of genes that are proximal in the linear order occurs on a similar scale to TADs. By promoting co-regulation of genes, TADs could explain the increased functional similarity between proximal genes.

We hypothesised that TADs form functional units and therefore genes within them are more likely to share functional annotations than can be explained by linear proximity alone. In order to test this hypothesis, we utilised some of the highest quality mammalian Hi-C data currently in the public domain [25] and annotated TADs using two TAD calling algorithms; Arrowhead and TopDom [23, 24]. We first assessed the relationship between TADs and gene paralogy as well as constraint. Then, focusing on TADs most likely to have been formed by loop extrusion involving CTCF, we assessed the functional relatedness of non-paralogous protein-coding genes within them, using four functional annotations: expression correlation, Gene Ontology (GO) semantic similarity, shared pathways and PPI.

## Results

### Characteristics of TADs in cortical neurons and embryonic stem cells (ESCs)

**Fig. 1:**
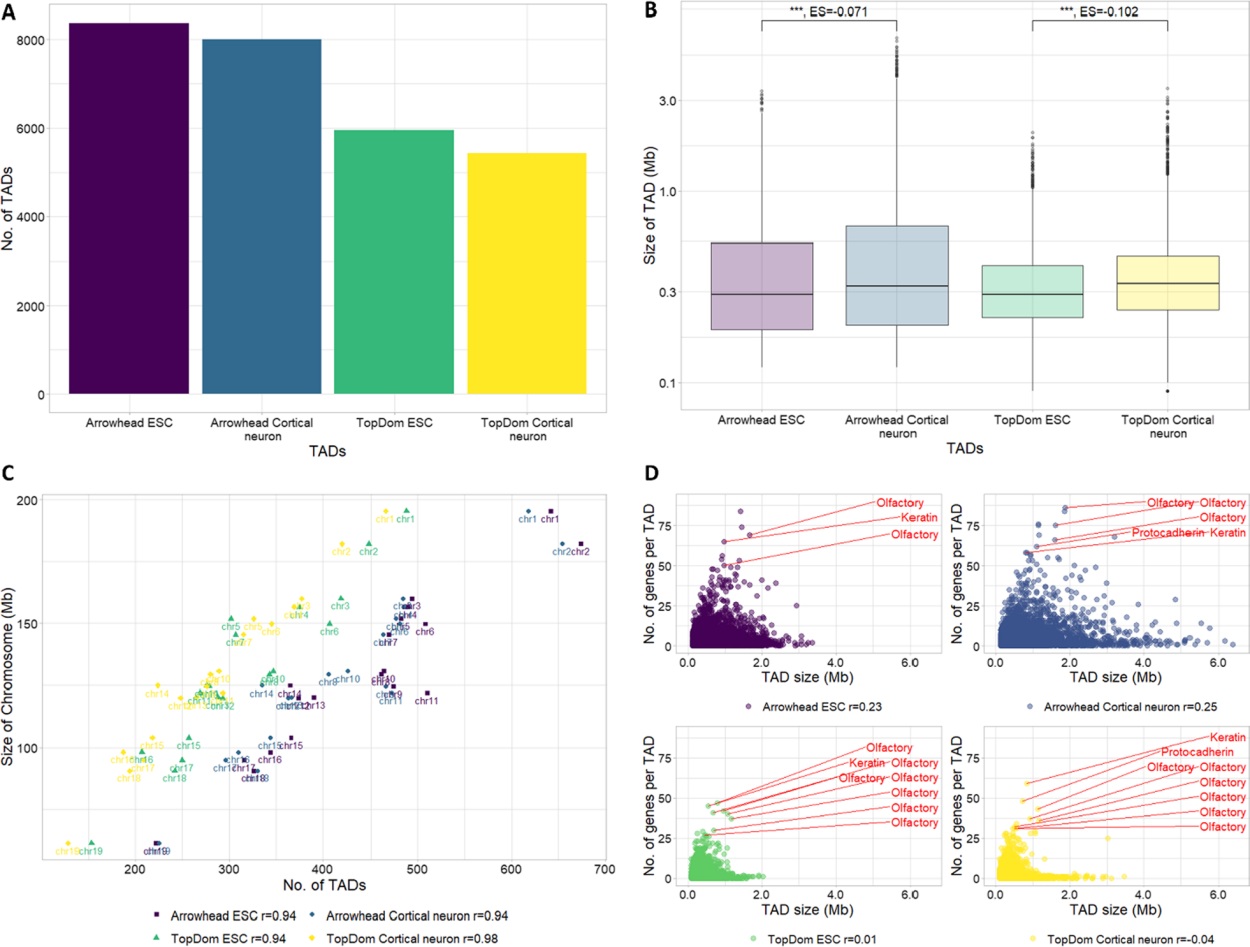
Features of autosomal TADs in ESCs and cortical neurons. **A** The number of TADs called using Arrowhead and TopDom in ESCs and cortical neurons. More TADs are called in ESCs than cortical neurons with both TAD callers. **B** Size of TADs called using Arrowhead and TopDom in ESCs and cortical neurons (plotted on a log_10_ scale). Both Arrowhead and TopDom call significantly smaller TADs in ESCs than cortical neurons (Wilcoxon test, *p*-value: *p* < 0.001 = ***, *p* < 0.01 = **, *p* < 0.05 = *, ES = effect size calculated using r for Wilcoxon). **C** The number of TADs per chromosome is strongly correlated with the size of the chromosome. **D** In both cell types and with both TAD callers most TADs have few genes. Overall, there is a low correlation between TAD size and gene number. Several TADs containing many genes were further investigated and found to contain multiple members of large gene families (annotated)

We analysed ESC and cortical neuron Hi-C data from Bonev et al. [25] using the Juicer pipeline [26]. This data represents some of the highest quality mammalian Hi-C data currently in the public domain. Throughout this work we have focused on autosomal TADs, so unless explicitly stated TADs refer to autosomal TADs only. We annotated 8371 (median size 0.29 Mb) and 5950 (median size 0.29 Mb) TADs in ESCs, and 8001 (median size 0.32 Mb) and 5430 (median size 0.33 Mb) TADs in cortical neurons, using two TAD callers Arrowhead [26] and TopDom [27], respectively (Fig. 1A–B). We detected similar sized TADs with Arrowhead and TopDom for both cell types. Our results confirm the finding from Bonev et al. (found using the directionality index TAD calling method) that there are more and smaller TADs in ESCs than cortical neurons [25].

To learn more about the distribution of TADs in the genome, we looked at their association with chromosomes and protein-coding genes, matching the expected null, we observed that, for both TAD callers and datasets, the number of TADs on a chromosome correlates strongly with the size of the chromosome (Fig. 1C) (*r* range 0.94 to 0.98).

Most TADs contain relatively few genes (mean number of genes: ~ 2.74 and ~ 3.49 (Arrowhead), and ~ 2.15 and ~ 2.62 (TopDom) in ESCs and cortical neurons, respectively) and there is little correlation between the number of genes within a TAD and the TAD size (*r* range −0.04 to 0.25) (Fig. 1D). We investigated several TADs which contained a large number of genes and found that they contained genes from large paralog families, e.g. olfactory genes and protocadherin (Fig. 1D). This is consistent with previous studies which have noted that genes from these functional families tend to fall within the same TAD, likely due to their shared regulatory requirements [12].

### TAD randomisation

**Fig. 2:**
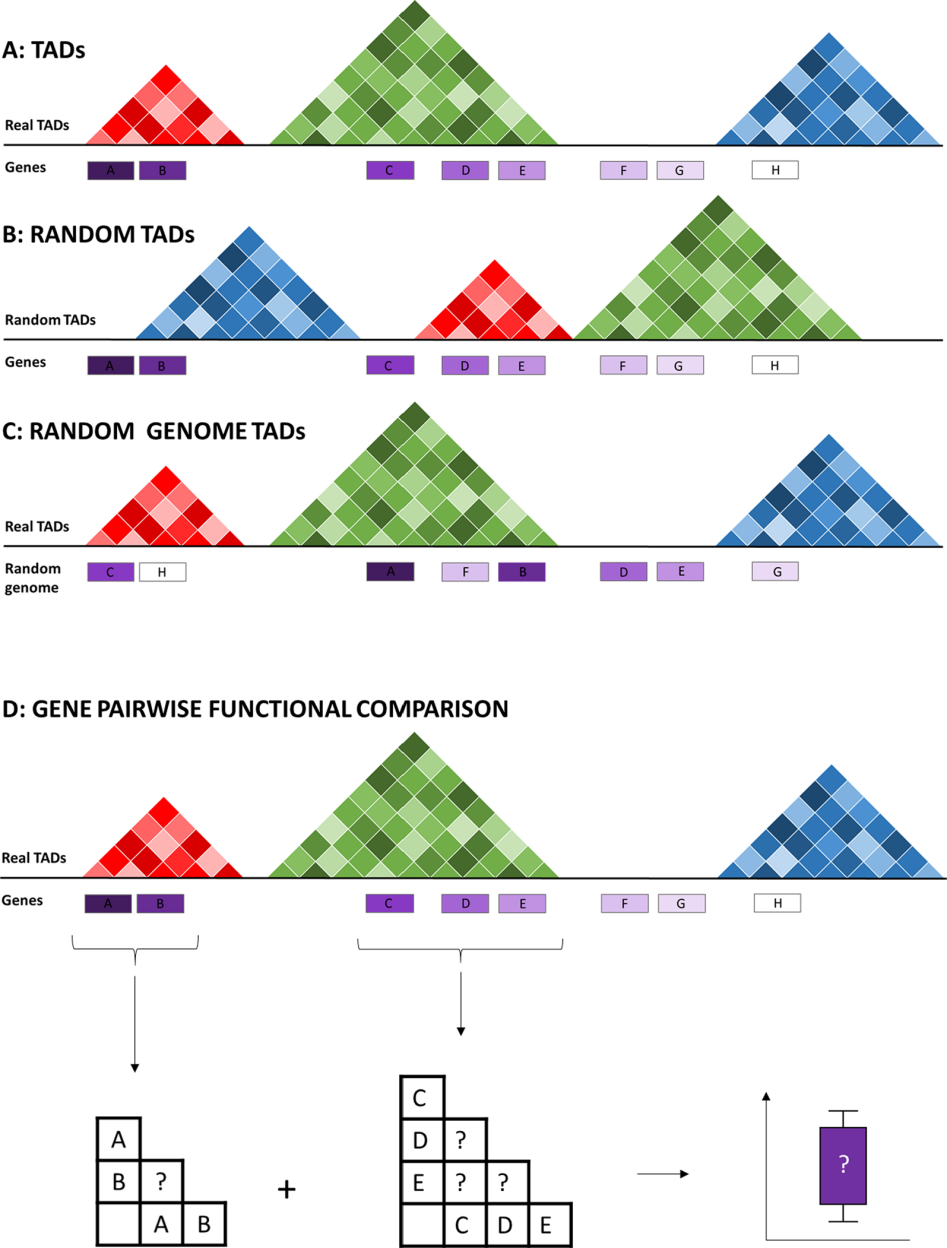
Randomisation and functional analysis procedure. **A** Schematic representing the structure of annotated TADs. **B** Null dataset one: random TADs. TADs were randomised within the same chromosome by selecting regions of equal size to the original TAD which also contain the same number of genes, thus controlling for the effect of the linear genome. **C** Null dataset two: random genome TADs. In order to remove the effect of the linear genome another null TAD set was generated in which the TADs remained in the same positions but the order of genes on the chromosome were randomised. **D** Pairwise strategy for comparing functional similarity between genes in the same TAD. All possible pairs of genes in each TAD were compared

**Fig. 3:**
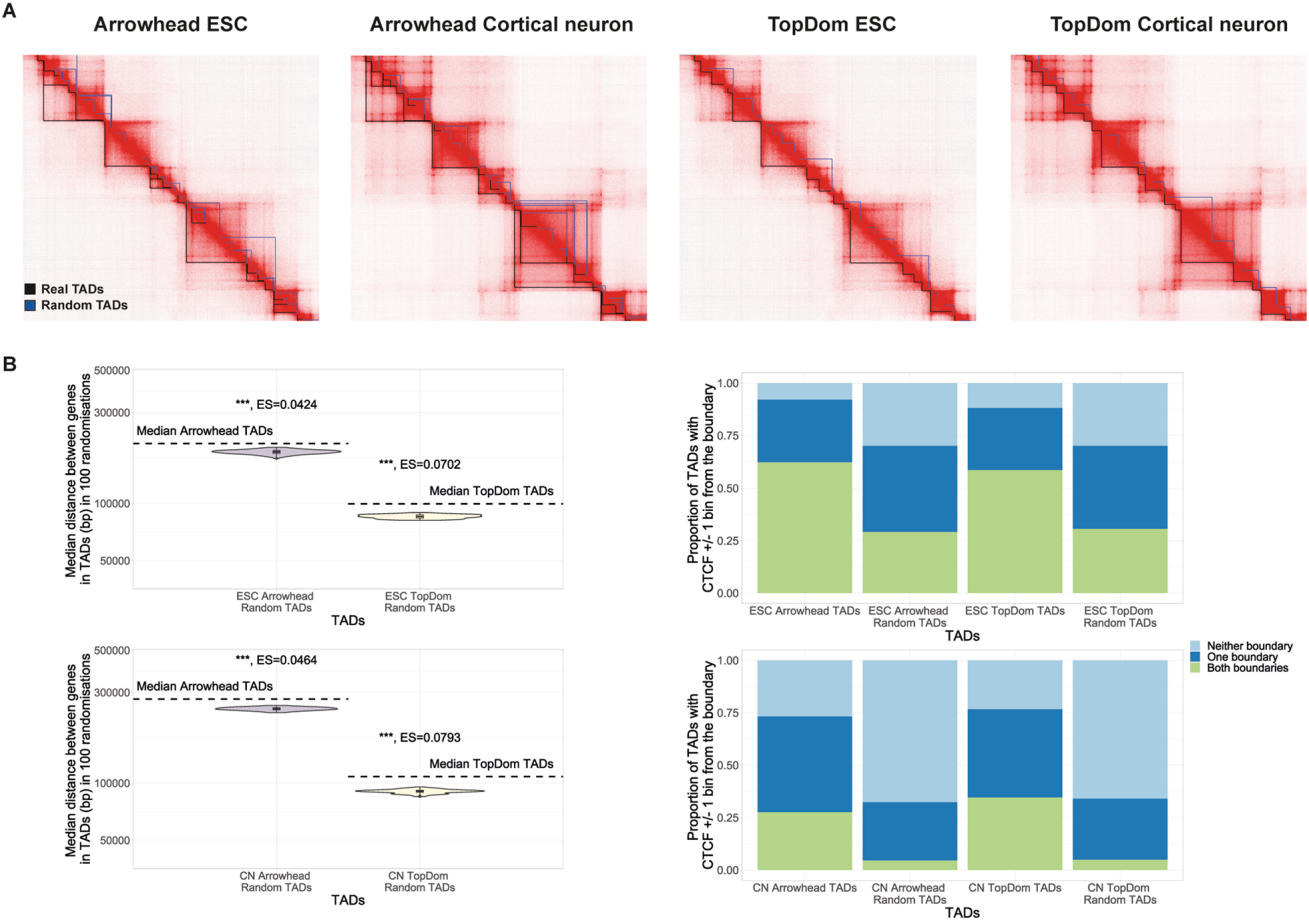
Features of autosomal TADs vs random TADs. **A** TADs (black) vs an example set of random TADs (blue) shown on the Hi-C matrix for the equivalent region of Chr2 in both ESCs and cortical neurons (CN). Matrices visualised using JuiceBox. **B** Median distance between gene start coordinates in TADs (dotted line) vs the median distance between genes in 100 sets of random TADs (plotted on a log_10_ scale). Genes are significantly closer together in random TADs than TADs (Wilcoxon test, median *p*-value: *p* < 0.001 = ***, *p* < 0.01 = **, *p* < 0.05 = *, ES = median effect size calculated using r for Wilcoxon). **C** Proportion of TADs with a CTCF binding site within ± 10 kb of both boundaries, one boundary or neither boundary. As expected a greater proportion of TADs have a CTCF binding site near both boundaries than in an example set of random TADs

In order to globally assess the functional similarity between genes in the same TADs, we sought to synthesise “random TADs” representing the null distribution. We developed two randomisation strategies in order to generate two null distributions controlling for different possible confounding signals. In the first randomisation strategy, which we refer to as random TADs, we maintained the basic structure of real TADs, i.e. TAD size, number of genes within the TAD and the approximate TAD overlap structure. This allowed us to control for the influence of linear gene order and distance which are known to correlate with gene functional similarity [23, 24]. In this randomisation strategy, the position of each TAD was randomised within the same chromosome to a new region of the same size, containing the same number of genes as the original TAD. For TopDom random TADs, TAD overlapping was prevented, reflecting the non-overlapping structure of TopDom TADs. For Arrowhead random TADs, if the new random TAD overlapped an existing random TAD this was controlled in order to favour “nested” TADs, thereby approximating the global TAD overlap structure seen in Arrowhead TADs (see “Methods”) (Figs. 2B, 3A). In the second randomisation method, which we refer to as random genome TADs, we again maintained the basic TAD structure but removed signal attributed to the linear gene. In order to do this, the positions of TADs were maintained but the identity of the genes in the genome were randomly shuffled within each chromosome (Fig. 2C). Using both randomisation strategies allows us to disentangle the functional similarity of genes within the same TAD from the functional similarity which can be attributed to proximity in the linear genome. Each TAD randomisation method was run 100 times for each cell type and each TAD caller.

In order to compare the functional similarity of genes within TADs to genes within random TADs or random genome TADs, we adopted a pairwise comparison approach (Fig. 2D). For every feature investigated, every possible pair of genes within a TAD/random TAD was compared in order to generate a distribution of scores. As TADs detected using Arrowhead can be nested or overlapping, the same pair of genes can exist in multiple Arrowhead TADs, in these cases gene pairs were considered only once. This means that for nested TADs (one TAD falls fully within another) pairs of genes from the largest of the nested TADs were considered and for overlapping TADs (only part of one TAD falls within another) pairs of genes from both TADs were considered with pairs of genes from the overlapping region considered once. The distribution of scores for each feature in TADs was compared to each of 100 sets of random TADs and the median *p*-value was reported.

To assess the gene distribution within TADs, we compared the distance between genes in TADs to genes in random TADs. We found that for both TAD callers, and both cell types, genes are significantly further apart in TADs than in random TADs (Fig. 3B, median *p*-value < 0.001).

It has previously been shown that TAD boundaries are enriched for CTCF which is hypothesised to play a crucial role in TAD formation by loop extrusion [5–8]. To assess this in our data we tested for the presence of CTCF ChIP-seq peaks near TAD boundaries vs random TAD boundaries (Fig. 3C, Additional file 1: Fig. S2). We observed that ~ 62%, 59%, 28%, 35% of ESC Arrowhead TADs, ESC TopDom TADs, cortical neuron Arrowhead TADs and cortical neuron TopDom TADs, respectively, had a CTCF ChIP-seq peak within in ± 10 kb of both TAD boundaries. This is compared to ~ 29%, 31%, 4.5%, 4.9% of ESC Arrowhead random TADs, ESC TopDom random TADs, cortical neuron Arrowhead random TADs and cortical neuron TopDom random TADs, respectively. Supporting previous reports [4, 6, 28, 29], this suggests that CTCF binding is common at the boundaries of TADs and is more prevalent than expected if TADs were randomly placed. This result also shows that more ESC TADs have a CTCF ChIP-seq peak near both boundaries than cortical neuron TADs. This could be due to a reduction in the number of chromatin domains formed by loop extrusion involving CTCF during differentiation. We noted that this still left 30%, 30%, 46% and 42% which had a CTCF ChIP-seq near only one boundary and 7.9%, 12%, 27% and 23% which did not have a ChIP-seq peak near either boundary, in ESC Arrowhead TADs, ESC TopDom TADs, cortical neuron Arrowhead TADs and cortical neuron TopDom TADs, respectively. This suggests that these domains may not be formed by loop extrusion involving CTCF and therefore may represent a different class of domains [11].

In order to assess the features of these TADs separately, we split TADs into CTCF TADs (which we define as TADs with a CTCF ChIP-seq peak within ± 10 kb of both boundaries) and nonCTCF TADs (which we define as TADs with a CTCF ChIP-seq peak within ± 10 kb of one, or neither boundary) (Additional file 1: Fig. S2). We compared the size of CTCF TADs and nonCTCF TADs between ESCs and cortical neurons. In both CTCF TADs and nonCTCF TADs we found cortical neuron TADs were significantly larger (*p*-value < 0.001, except Arrowhead nonCTCF TADs which was not significantly different) (Additional file 1: Fig. S3A–B). We also compared the distance between genes in CTCF and nonCTCF TADs to random CTCF/nonCTCF TADs, respectively. We found that genes are significantly further apart in both CTCF TADs and nonCTCF TADs than expected in random CTCF/nonCTCF TADs (median *p*-value < 0.001) (Additional file 1: Fig. S3C–D).

To further investigate the biological context of CTCF TADs and nonCTCF TADs we calculated the percentage of TADs which were CTCF TADs or nonCTCF TADs, and in the A or B compartments (Additional file 2: Table S4). We found that for both TAD callers and tissues CTCF TADs were most commonly in the A compartment (~ 44%, 40%, 20% and 23% of TADs are CTCF TADs and in the A compartment compared to ~ 18%, 18%, 7% and 12% of TAD which are CTCF TADs and in the B compartment in ESC Arrowhead TADs, ESC TopDom TADs, cortical neuron Arrowhead TADs and cortical neuron TopDom TADs, respectively). Whereas ESC nonCTCF TADs are more commonly found in the B compartment (percentage of TADs which are nonCTCF in compartment A vs B in ESC Arrowhead TADs: ~ 11% vs 26%, ESC TopDom TADs: ~ 8% vs 33%) and cortical neuron nonCTCF TADs are split relatively equally between A and B compartments (cortical neuron Arrowhead TADs: ~ 42% vs 31% and cortical neuron TopDom TADs: ~ 30% vs 35%).

### TADs vs paralogy and gene constraint

**Fig. 4:**
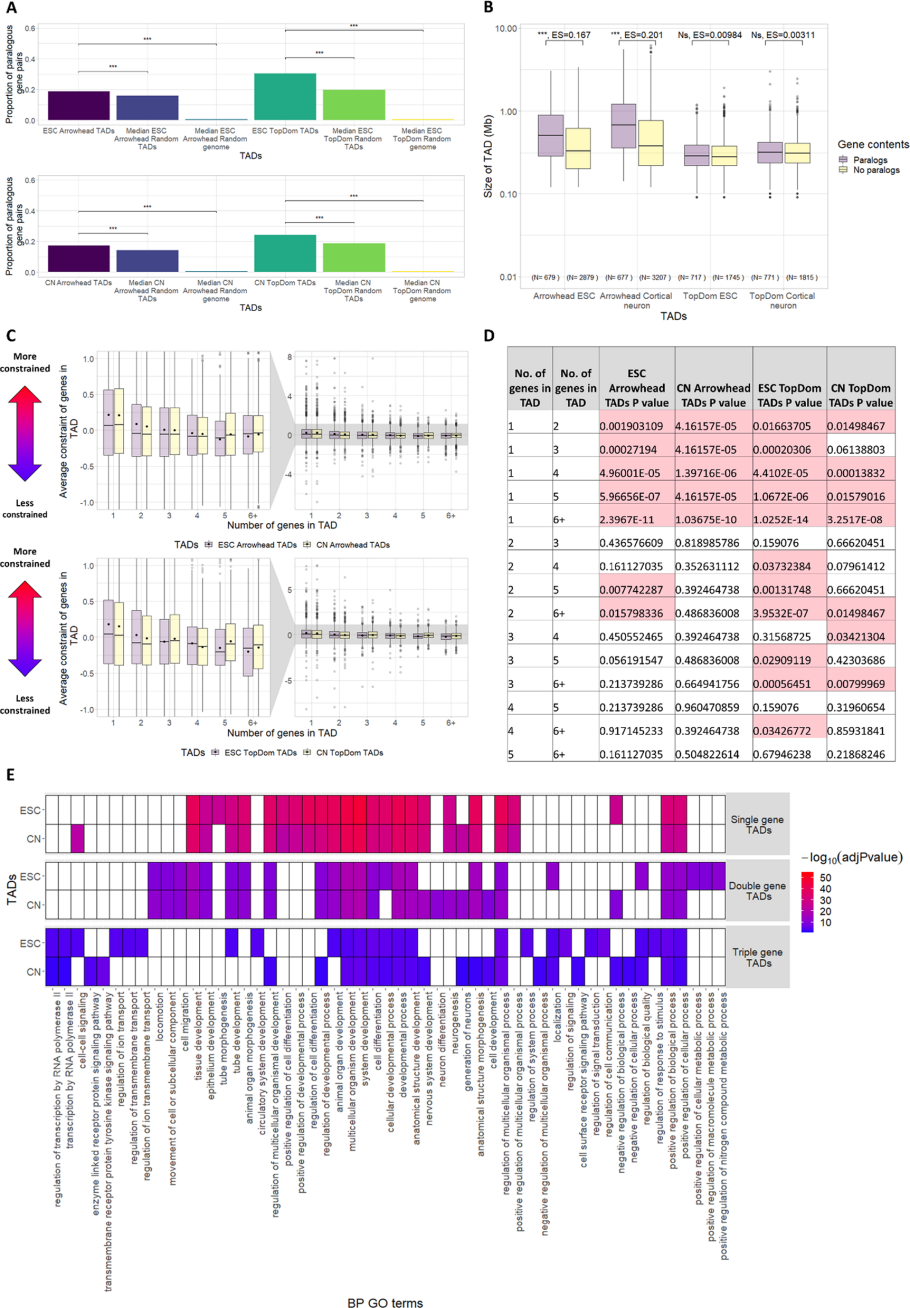
Paralogs and constraint vs autosomal TADs. **A** Proportion of paralogous gene pairs in TADs, the median proportion in 100 sets of random TADs, and the median proportion in 100 sets of random genome TADs. TADs contain significantly more pairs of paralogous genes than both random TADs and random genome TADs (Fisher’s exact test, median *p*-value: *p* < 0.001 = ***, *p* < 0.01 = **, *p* < 0.05 = *). **B** Size of TADs containing pairs of paralogs vs TADs (with > 1 gene) containing no pairs of paralogs (plotted on a log_10_ scale). For Arrowhead TADs, TADs which contain pairs of paralogs are larger than TADs which have no paralog pairs. (Wilcoxon test, *p*-value: *p* < 0.001 = ***, *p* < 0.01 = **, *p* < 0.05 = *, ES = effect size calculated using r for Wilcoxon). **C** Distribution of mean constraint scores of genes occupying the same TAD. TADs are split depending on the number of genes they contain (1, 2, 3, 4, 5, 6+). Dots indicate the mean of the distribution. **D** Table showing FDR corrected p-values of differences between groups in C calculated with the Wilcoxon test. Significant *p*-values are highlighted red. Genes singly occupying a TAD have a significantly higher constraint score than the average constraint of genes in TADs with > 1 gene. **E** Biological processes GO term functional enrichment of genes singly, doubly or triply occupying an Arrowhead TAD. Only the top 25 most significant GO terms passing a *p*-value threshold of < 0.05 (multiple testing corrected using the “gSCS” option) are shown for each gene set

We have shown examples of TADs that contain a large number of genes from the same paralogous families (Fig. 1D), suggesting that genes within TADs could be more functionally similar due to shared ancestry [30]. We therefore investigated whether genes within TADs are enriched for paralogous gene pairs, genome wide. To do this we assessed the proportion of paralogous gene pairs within TADs and random TADs. Similarly to Ibn-Salem et al. [31] we found a greater proportion of paralogous gene pairs fall within TADs compared to random TADs (Fig. 4A, median *p*-value of TADs vs random TADs < 0.001). This suggests that pairs of paralogous genes are more likely to fall within the same TAD than can be explained by the linear proximity of the genes alone. We further investigated this relationship and found that Arrowhead TADs which contain at least one pair of paralogs are significantly larger in size than TADs with no pairs of paralogs (Fig. 4B, *p*-value < 0.001). However, no difference in size was observed between TopDom TADs containing at least one pair of paralogs or no pairs of paralogs. Despite this both Arrowhead and TopDom TADs containing a pair of paralogs were significantly larger than observed in random TADs containing a pair of paralogs, and significantly smaller than random genome TADs containing a pair of paralogs (Additional file 1: Fig. S4A) (median *p*-value < 0.01). This suggests that TADs containing a pair of paralogs are significantly larger than expected if TADs are randomly placed in the genome and significantly smaller than expected if the effect of the linear order of the genome is randomised (the latter result is probably due to the increased probability of larger TADs containing a pair of paralogs in a randomised genome). No significant difference was observed between the size of TADs and random TADs which contained no pairs of paralogs.

When TADs are split into CTCF TADs and nonCTCF TADs we find that pairs of paralogs are significantly enriched in nonCTCF TADs compared to random nonCTCF TADs (median *p*-value of nonCTCF TADs vs random nonCTCF TADs < 0.001). However, on the whole this is not true for CTCF TADs where paralogous pairs are largely depleted (with the exception of ESC TopDom TADs) (median *p*-value of CTCF TADs vs random CTCF TADs < 0.001 for ESC Arrowhead and cortical neuron Arrowhead and < 0.05 for cortical neuron TopDom) (Additional file 1: Fig. S5). This suggests that although pairs of paralogs are enriched in TADs they are largely depleted in CTCF TADs. This raises the possibility that TADs detected by Arrowhead and TopDom may be made up of two functional groups. As CTCF TADs are bounded by CTCF, they are likely to have been formed by loop extrusion involving CTCF. In contrast, nonCTCF TADs may have been formed by other mechanisms and therefore may represent other types of domain, e.g. compartment domains or TADs formed by loop extrusion not involving CTCF [10].

In order to further assess the impact of evolutionary forces on genes within TADs, we assessed the average constraint scores of genes in TADs. Constraint scores quantify the degree of selective constraint acting on protein-coding genes, with a higher score indicating a greater strength of purifying selection [32]. Selective constraint can change over evolutionary time, and we therefore considered constraint scores calculated in the mouse lineage [33]. We find that protein-coding genes which singly occupy a TAD are significantly more constrained than the mean constraint of genes co-occupying TADs (Fig. 4C–D, Additional file 2: Table S1). Genes singly occupying a TAD also have significantly higher constraint than seen in random TADs (with the exception of cortical neuron TopDom random TADs) suggesting the result cannot be explained by the structure of the linear genome alone. Genes singly occupying a TAD are also significantly more constrained than seen in random genome TADs (FDR corrected median *p*-value of genes singly occupying TADs vs genes singly occupying TADs in random TADs < 0.05 or random genome TADs < 0.001, Additional file 1: Fig. S6). This suggests that genes, which singly occupy TADs, may be under higher selective constraint and may be more functionally important than genes which co-occupy a TAD. This in turn suggests that the protection from aberrant regulation of functionally important genes, implied by being in a private TAD, is under selective constraint.

We next sought to test if the relationship between TADs and average gene constraint is observable in both CTCF TADs and nonCTCF TADs. When considering either CTCF TADs or nonCTCF TADs, as seen above, we find that generally the constraint of genes in singly occupied TADs is significantly higher relative to the average constraint of genes co-occupying a CTCF/nonCTCF TAD (Additional file 1: Fig. S7, Additional file 2: Tables S2 and S3).

In order to assess which biological processes genes which singly occupy a TAD are involved in, we carried out a functional enrichment analysis (see “Methods”) using Biological process GO terms (Fig. 4E and Additional file 1: Fig. S8). We found that genes which singly occupy a TAD are highly enriched for developmental processes, genes which occupy a TAD with one other gene (double occupancy) are also enriched for developmental processes but to a lesser extent and genes which occupy a TAD with two other genes (triple occupancy) are less enriched for developmental processes still. For example, “system development” is the most significant GO term associated with singly occupied Arrowhead TADs in both ESCs and cortical neurons (*p*-value = 8.96 × 10^–48^ and 4.02 × 10^–43^, respectively), but it is less significantly associated in doubly occupied or triply occupied TADs (doubly occupied: *p*-value = 5.31 × 10^–20^ and 3.11 × 10^–20^, triply occupied: *p*-value = 9.30 × 10^–06^ and 1.35 × 10^–06^ for ESC and cortical neuron, respectively). We repeated the enrichment analysis using genes which singly, doubly and triply occupy random TADs in order to establish whether randomly placed TADs with similar features (e.g. only one gene in the length of the TAD) have a similar pattern of enrichment (Additional file 1: Fig. S8). We found that genes that singly occupy a random TAD are also enriched for developmental processes but to a lesser degree than TADs. This suggests that the enrichment for developmental function observed in genes that singly occupy a TAD cannot be explained by the linear genome alone.

In order to further investigate the similarity of sets of genes singly occupying TADs between cell types, we compared genes singly occupying a TAD in ESC with cortical neuron. We found that for both Arrowhead and TopDom, genes which singly occupied TADs were very similar between ESC and cortical neuron (~ 64% and ~ 58% of ESC and ~ 68% and ~ 61% of cortical neuron genes singly occupying TADs were also singly occupying TADs in the other tissue for Arrowhead and TopDom, respectively) (Additional file 1: Fig. S9A). We next tested the functional enrichment of the genes singly occupying TADs in: ESC only, cortical neuron only or both ESC and cortical neuron. In general we found enrichment for similar developmental functions between the groups (Additional file 1: Fig. S9B). Together these results suggest that similar genes singly occupy TADs in both tissues.

We also assessed singly occupied TADs in the context of their compartment and CTCF/nonCTCF TAD identity (Additional file 2: Table S5). We found that ESC singly occupied TADs were most commonly CTCF TADs in the A compartment. Suggesting that they are more likely to be transcriptionally active and formed by loop extrusion involving CTCF. On the other hand cortical neuron singly occupied TADs were most commonly nonCTCF TADs with similar proportions in the A and B compartments.

### Expression and functional similarity of non-paralogous genes in CTCF TADs

We have shown that CTCF TADs and nonCTCF TADs are unequal in their functional relevance, with nonCTCF TADs enriched for paralogous gene pairs. We therefore next focused on the functional similarity of pairs of genes in CTCF TADs (Fig. 2). Since paralogous gene pairs are highly likely to share functional similarity and we have previously assessed their relationship with TADs (Fig. 4A–B, Additional file 1: Fig. S5) we excluded all pairs of paralogous genes and removed the olfactory genes (see “Methods”) in all functional analyses. This will allow the assessment of functional similarity between genes within TADs without recent shared ancestry.

**Fig. 5:**
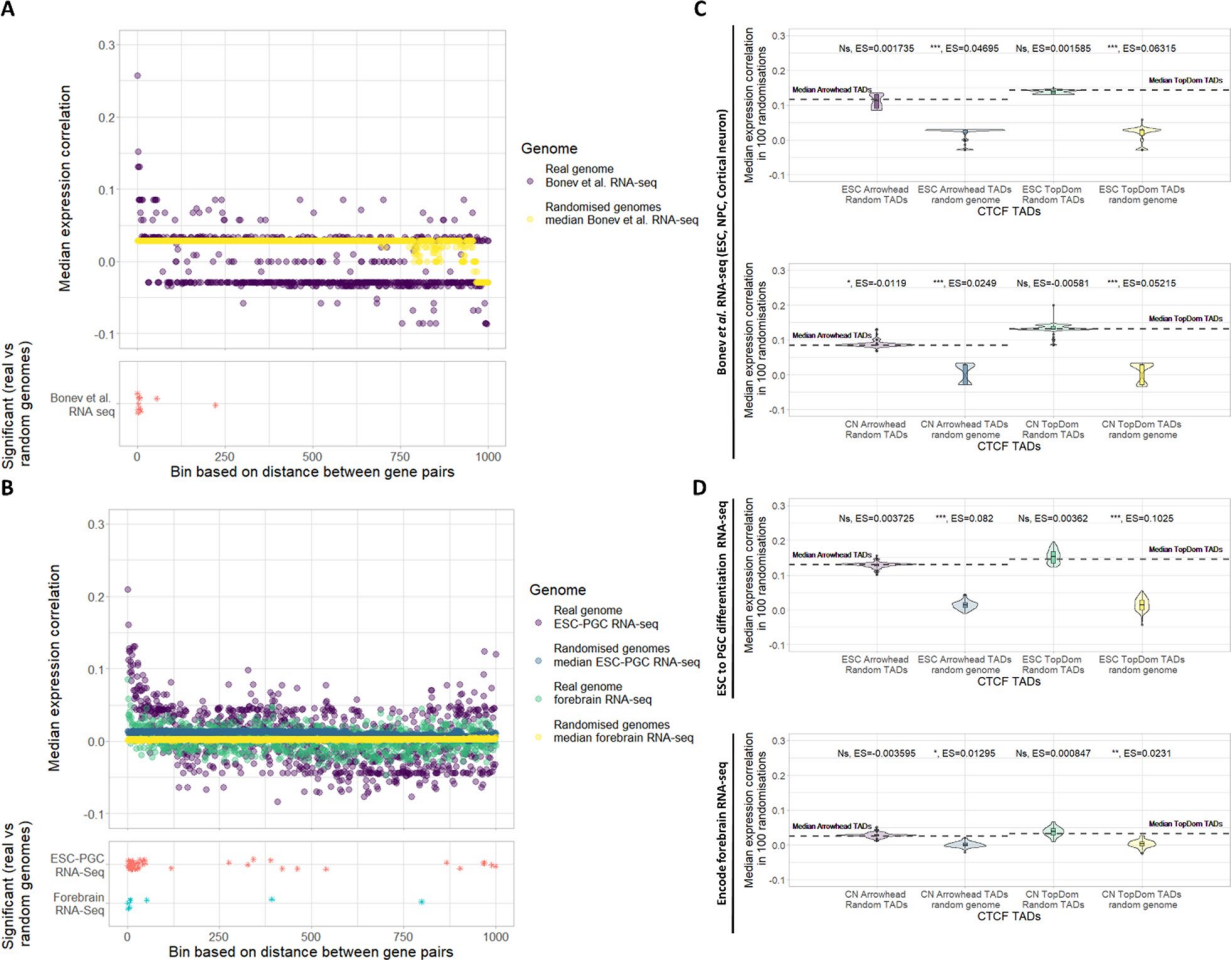
Pairwise gene co-expression in autosomal CTCF TADs. Olfactory genes and paralogous gene pairs have been excluded in all panels. **A**–**B** Top panel: median expression correlation coefficient (spearman) for pairs of genes vs binned distance in the real genome and 1000 random genomes. Bottom panel: stars indicate bins with a significantly higher median expression correlation in the real genome vs 1000 random genomes (FDR corrected *p*-value < 0.05). Jitter has been applied on the y axis to allow clearer visualisation of close together points. **A** Expression correlation coefficients were calculated using RNA-seq from two replicates each of ESC, neural progenitor cells (NPC) and cortical neuron cells. **B** Expression correlation coefficients were generated using mouse ESCs differentiating to primordial germ like cells (PGC) and forebrain RNA-seq from encode. The mouse ESCs differentiating to PGC RNA-seq was generated with three replicates each of ESCs, epiblast like cells (day 2), PGC (day 4) and PGC (day 6). The forebrain RNA-seq was generated with two replicates each, of embryos of varying ages. **C**–**D** Median expression correlation coefficient (spearman) for pairs of genes in CTCF TADs (dotted line) and median expression correlation coefficient (spearman) in 100 sets each of: random CTCF TADs and random genome CTCF TADs. CTCF TADs called using both Arrowhead and TopDom, in both ESC and cortical neuron (CN) Hi-C (Wilcoxon test, median *p*-value: *p* < 0.001 = ***, *p* < 0.01 = **, *p* < 0.05 = *, NS = not significant, median ES = effect size calculated using r for Wilcoxon). **C** Expression correlation coefficients were calculated using RNA-seq from A. **D** Expression correlation coefficients were calculated using RNA-seq from B

In order to assess whether pairs of genes in the same TAD have correlated expression patterns we used FPKM/RPKM counts from RNA-seq expression data. RNA-seq generated during neural differentiation from Bonev et al. [25] and from the most closely matching cell types/tissues to ESCs and cortical neurons which had greater than three samples (mouse ESCs differentiating to primordial germ cell like cells (PGC) and forebrain at different embryonic stages, respectively) from Encode or GEO were used [34–37]. Using these expression counts we calculated spearman’s rank correlation coefficient between pairs of genes in CTCF TADs, 100 sets of random CTCF TADs, and 100 sets of random genome CTCF TADs (Fig. 5C–D). We found pairs of genes in CTCF TADs have a significantly higher expression correlation than pairs of genes in random genome CTCF TADs in all comparisons (median *p*-value < 0.05). This is an expected result because randomising the genome removes the effect of linear gene proximity. However, in 7 out of 8 comparisons we find no significant difference in expression correlation between pairs of genes in CTCF TADs and pairs of genes in random CTCF TADs (median *p*-value < 0.05). This suggests that contrary to the majority of other studies [17–20] we find little evidence that pairs of genes sharing a TAD are more likely to have similar expression patterns than can be explained by their linear proximity. A study by Soler-Oliva et al. found that algorithmically identified co-expression domains in breast tissue/breast cancer tend not to coincide with TADs, which supports our findings [38].

**Fig. 6:**
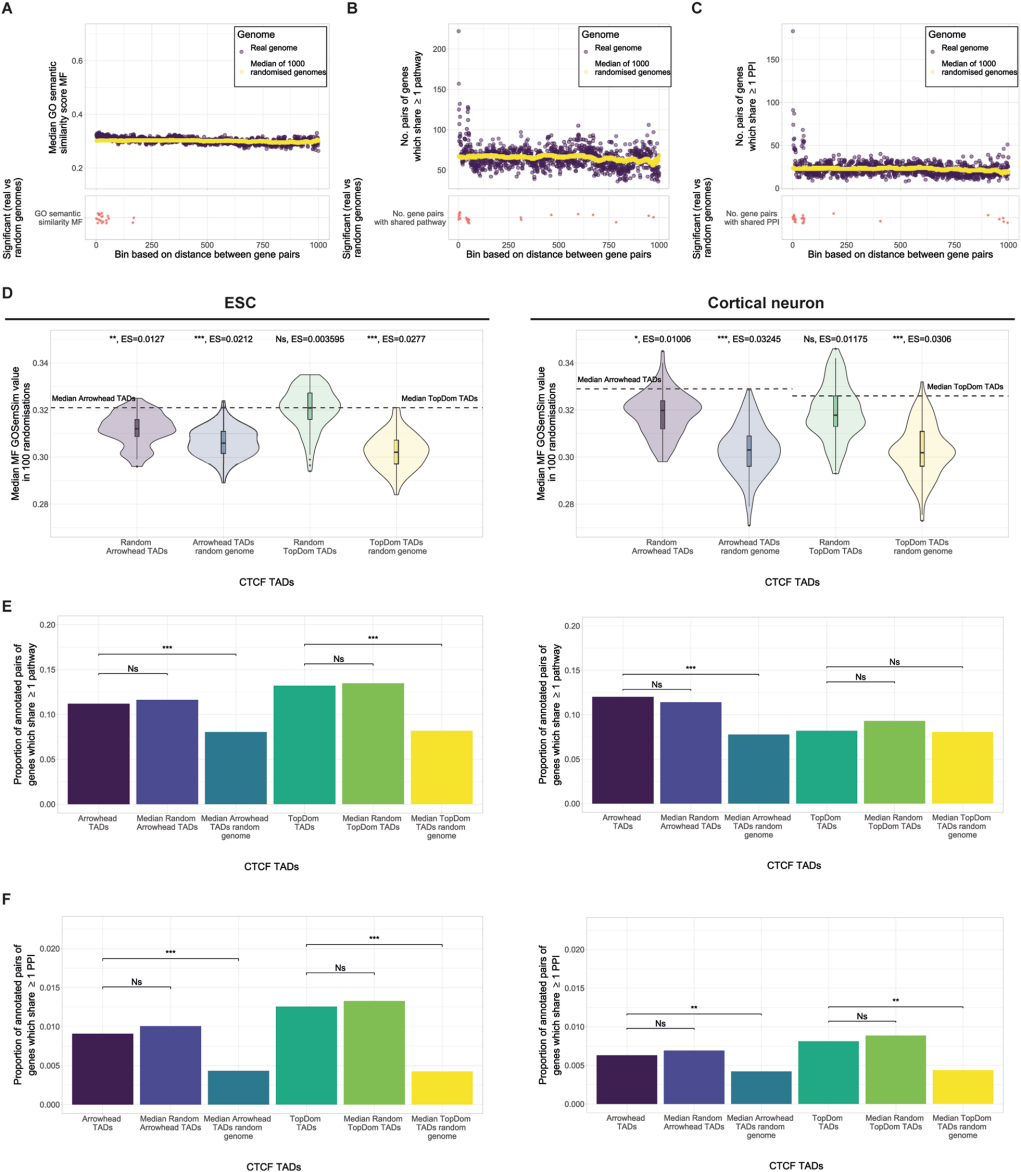
Functional similarity of pairs of genes in autosomal CTCF TADs. Olfactory genes and paralogous gene pairs have been excluded in all panels. **A**–**C** Top panel: median GO semantic similarity, number of gene pairs with ≥ 1 shared pathways or number of gene pairs with ≥ 1 shared PPI for pairs of genes vs binned distance in the genome and 1000 random genomes. Bottom panel: stars indicate bins with a significantly higher functional similarity in the genome vs 1000 random genomes (FDR corrected *p*-value < 0.05). **D**–**F** TADs called using both Arrowhead and TopDom; in both ESC and cortical neuron Hi-C. Median *p*-value: *p* < 0.001 = ***, *p* < 0.01 = **, *p* < 0.05 = *, NS = not significant. **A** Distribution of MF GO semantic similarity for pairs of genes binned by distance in the genome vs 1000 random genomes. **B** Distribution of the number of pairs of genes sharing ≥ 1 pathway binned by distance in the genome vs 1000 random genomes. **C** Distribution of the number of pairs of genes sharing ≥ 1 PPI binned by distance in the genome vs 1000 random genomes. **D** Median MF GO semantic similarity for pairs of genes in CTCF TADs (dotted line) compared to the distributions of median MF semantic similarity for 100 sets each of: random CTCF TADs and random genome CTCF TADs (Wilcoxon test, median *p*-value, median ES = effect size calculated using r for Wilcoxon). **E** Proportion of annotated pairs of genes sharing ≥ 1 pathway in CTCF TADs and the median proportion of pairs of genes sharing ≥ 1 pathway in 100 sets each of: random CTCF TADs and random genome CTCF TADs (Fisher’s exact test, median *p*-value). **F** Proportion of annotated pairs of genes sharing ≥ 1 PPI in CTCF TADs and the median proportion of pairs of genes sharing ≥ 1 PPI in 100 sets each of: random CTCF TADs and random genome CTCF TADs (Fisher’s exact test, median *p*-value)

Next, we sought to assess whether pairs of genes within the same CTCF TAD are more likely to share functional annotations than pairs of genes in random CTCF TADs or random genome CTCF TADs. Here, we used molecular function (MF) GO semantic similarity scores, shared pathways, and PPI (see “Methods”). In 11 out of 12 comparisons we found that pairs of genes in CTCF TADs are significantly more similar (median *p*-value < 0.01) in terms of functional annotation than pairs of genes in random genome CTCF TADs (Fig. 6D–F). Again, this is expected as randomising the genome removes functional similarity that can be explained by linear proximity. Using binned linear distance, we found greater similarity between pairs of genes which are in very close linear proximity than expected if genes were randomly ordered on the chromosome (Fig. 6A–C). We next compared the functional annotations of genes in CTCF TADs with genes in random CTCF TADs. We found that for the majority of comparisons there was no significant difference (10 out of 12 comparisons, median *p*-value < 0.05) (Fig. 6D–F). Pairs of genes in Arrowhead ESC CTCF TADs and Arrowhead cortical neuron CTCF TADs have significantly more similar MF GO terms than pairs of genes in random CTCF TADs (median *p*-value < 0.01 and < 0.05, respectively). A similar trend in MF GO similarity was observed for all other CTCF TADs compared to random CTCF TADs but the difference wasn’t significant in the other 2 comparisons (median *p*-value < 0.05). This could indicate that pairs of genes in CTCF TADs have slightly more similar MF GO term annotations than pairs of genes in random CTCF TADs. However, perhaps this is limited to few TADs as the increase in similarity is very small and often not significant. Overall, we find the biggest contribution to the functional similarity between pairs of genes in CTCF TADs can be attributed to their linear proximity in the genome. When we control for linear proximity we find a less consistent picture but in the majority of comparisons, pairs of genes in CTCF TADs are no more likely to be functionally similar than if CTCF TADs were randomly placed.

## Discussion

TADs are thought to co-localise regulatory elements and their target genes, insulate genes from off-target enhancer interactions, and block the spread of genome activation [12]. Due to these findings we hypothesised that genes sharing a TAD would be more likely to be co-regulated. This is because in the absence of further insulation/specificity, enhancers may be able to “scan” all regulatory elements in the TAD. We hypothesised that if this is the case one might expect genes within TADs to have higher co-expression and greater functional similarity than can be explained purely by the proximity of genes in the linear order of the genome.

Similarly to previously described work by Ibn-Salem et al. [31] we found that pairs of paralogous genes are more likely to fall within the same TAD than expected if TADs are randomly placed within chromosomes (Fig. 4A). This presents a clear case in which TADs contain functionally similar genes and could reflect the need for paralogs to share regulatory elements. However, we go on to show that TADs can be split into CTCF TADs and nonCTCF TADs, and whilst pairs of paralogous genes are more likely to fall within nonCTCF TADs than randomly placed nonCTCF TADs, paralogous gene pairs are commonly depleted in CTCF TADs (Additional file 1: Fig. S5). This suggests that paralogs are depleted in domains formed by loop extrusion involving CTCF.

To further investigate the relationship between gene evolution and TADs, we analysed the average gene constraint of genes sharing TADs. We found that genes which singly occupy a TAD are statistically more constrained than genes in TADs with multiple genes. This suggests that genes in TADs on their own are less tolerant to mutation and therefore more functionally important. This is supported by Muro et al. [39] who recently found that genes which singly occupy a TAD are more likely to be associated with disease. We analysed the functional enrichment of the genes singly occupying TADs and found a strong enrichment for developmental processes. A similar result was also reported in a parallel effort by Wu et al. who found that developmental genes are topologically isolated in CTCF “loop domains” in human pluripotent stem cells [40]. Together these results indicate that there is a selective pressure for functionally important developmental genes to fall privately within TADs providing them with strong insulation from aberrant regulation.

Our results indicate that there is little global evidence for an increase in expression correlation or functional annotation similarity in genes sharing a TAD. In general, we found no difference in expression correlation between pairs of non-paralogous protein-coding genes in CTCF TADs vs random CTCF TADs. This is contrary to previous findings [17–20]. We also found that pairs of non-paralogous protein-coding genes within CTCF TADs are largely not more similar in functional annotation than in random TADs. This suggests that globally TADs are not associated with a higher degree of co-regulation between the genes they contain. However, there may be instances of individual TADs containing co-regulated/functionally similar genes on a local scale.

We postulate that CTCF TADs are likely to have been formed by loop extrusion involving CTCF. However, the mechanism involved in the formation of nonCTCF TADs is unclear, therefore these domains may represent other domain categories, e.g. compartmental domains or TADs formed by loop extrusion not involving CTCF. The results presented here support the assertion made by Beagan et al. [11] that it is important to separate domains formed by different mechanisms because they are likely to have different functional properties.

It is widely reported that TAD calling is highly variable [41–43], in order to mitigate against this, we have included results from two TAD callers, Arrowhead and TopDom. Many of the conclusions reported here are largely consistent across the two TAD callers. Where disagreements exist this is likely due to differences in the TAD calling methods and highlights the importance of ensuring findings are robust to the choice of TAD caller. TopDom annotates the entire genome with TADs therefore if regions exist in the genome which have no TADs, TopDom will still attempt to call them, this could increase noise in TADs called by TopDom by the inclusion of false positives. On the other hand Arrowhead calls TADs sporadically and therefore could be prone to false negatives. In the future the field would greatly benefit from efforts to develop a gold standard TAD calling method.

Together our results suggest TADs play a stronger role in insulating genes from aberrant regulation rather than promoting co-expression of genes within a TAD. We speculate that our results are more compatible with a model of TADs in which enhancers are prevented from interacting with all the genes within the same TAD by evolved enhancer-promoter specificity or further insulation in the form of tissue/cell type specific sub-TADs. We also noted that genes which share a TAD are significantly further apart in linear distance than expected if TADs are randomly placed. Therefore, perhaps one such mechanism by which enhancer-promoter specificity is maintained within TADs is by selection pressure for genes in TADs to be further apart in linear distance. If this is the case, disease associated variants within a TAD may have deleterious consequences by misregulating their normal target gene, but also may acquire gain-of-function regulation of other genes within the TAD.

## Conclusions

Our results suggest a limited role for TADs in promoting co-regulation of the genes within them. We find evidence that pairs of paralogous genes fall within TADs more often than random TADs. However, we find that pairs of paralogous genes are only enriched in nonCTCF TADs. The functional differences observed between CTCF and nonCTCF TADs may reflect the possibility that nonCTCF TADs are more similar to other types of chromatin domain (e.g. compartmental domains) than TADs or that they constitute a functionally distinct class of TADs. We find little evidence that non-paralogous protein-coding genes within the same CTCF TAD are more likely to have correlated expression patterns or similar functional annotations than non-paralogous protein-coding genes in random TADs. This suggests that TADs formed by loop extrusion involving CTCF do not have a global association with co-regulation and the formation of “gene regulatory domains”. We find evidence that genes that singly occupy a TAD have significantly higher constraint. This suggests that these genes may be more functionally important and TADs may be acting to insulate them from aberrant regulation. Overall, our results suggest a stronger role of TADs in regulatory insulation than promotion of co-regulation.

## Methods

### Topologically associating domains

Mouse ESC and cortical neuron Hi-C data published in [25] was downloaded from Gene Expression Omnibus (GEO) (Accession Number: GSE96107). These datasets represent two of the high-resolution mammalian Hi-C datasets published to date. Hi-C data were analysed using the Juicer analysis pipeline aligning to the mm10 genome build [26]. Parameters within Juicer were selected so that contacts with a mapping quality (MAPQ) below 30 were filtered. For each cell type all replicates were run through the Juicer pipeline separately and were combined using the “mega” option in Juicer. The map resolution (minimum bin size at which 80% of bins have over 1000 contacts), which is commonly used to specify the minimum bin size at which a Hi-C dataset should be analysed was 950 bp and 750 bp for ESC and cortical neuron, respectively. Hi-C data were binned at 10 kb and Vanilla coverage (VC) normalisation was employed (as it was compatible with both Arrowhead and TopDom). The sex chromosomes were excluded from the analysis throughout.

It has been shown that algorithmically determined TADs can vary widely depending on the TAD caller used [41, 42, 44]. In order to make sure that results are robust to the choice of TAD caller, TADs were called using Arrowhead and TopDom at 10 kb. Arrowhead TADs were called with default settings (*m* = 2000). TopDom TADs were called using the parameter *w* = 20, as this was deemed to be an appropriate setting to identify TADs at 10 kb (Additional file 1: Fig. S12). TADs detected by Arrowhead ranged in size from 120 Kb to 6.38 Mb and TopDom TADs ranged in size from 10 kb to 3.47 Mb. As TADs with a length constituting only several genomic bins are unlikely to be real we filtered out TopDom TADs < 90 kb. Arrowhead calls overlapping TADs. Whereas TopDom calls non-overlapping regions annotated “domain”, “boundary” or “gap”, similarly to Dali et al. only “domain” annotations were considered in this work [42]. TADs were called on Hi-C maps made from merged replicates. Between 21.38% and 45.5% of TADs called by one TAD caller had an equivalent TAD (within ± 2bins at both boundaries) called by the other TAD caller (Additional file 2: Table S6).

It is widely suggested that TADs are formed by a loop extrusion process involving convergent CTCF bound at TAD boundaries and cohesin [11]. Where indicated, TADs have been split into CTCF TAD or nonCTCF TADs. In order to do this ESC and cortical neuron CTCF ChIP-seq peaks (generated alongside the Hi-C data [25]) were downloaded from GEO (GEO Accession Number: GSE96107). TADs where both boundaries were within ± 1 bin (10 kb) of a CTCF peak were considered to be “CTCF TADs”, the equivalent TADs in random TADs or random genome TADs were used for comparison. Whereas, TADs with only one boundary or neither boundary within ± 1 bin (10 kb) of a CTCF peak were considered to be “nonCTCF TADs”. Between 23.74 and 45.99% of CTCF TADs and 15.13 and 28.72% of nonCTCF TADs called by one TAD caller had an equivalent CTCF/nonCTCF TAD (within ± 2bins at both boundaries) called by the other TAD caller (Additional file 2: Table S7).

### Compartments

Compartments were identified after filtering contacts with MAPQ below 30, at 50 kb bin size and VC normalisation using the eigenvector function in Juicer [26]. The output from Juicer was then processed as outlined in Miura et al. [45].

### Overlapping TADs with protein-coding genes

Ensembl IDs of mouse protein-coding genes and mm10 coordinates were downloaded from BioMart (release 96) [46]. Using bedtools intersect [47], protein-coding genes were overlapped and assigned to a TAD if their start and end position fell within the same TAD. This TAD-gene mapping method is more stringent than previously used [17, 18, 39], but it allows us to focus on genes which can be confidently assigned to a TAD and controls for the possibility that genes which overlap a TAD boundary may have different features. This is especially important given recent evidence has shown that TAD boundaries are often not “sharp”, instead boundaries can span “zones of transition” meaning that it may not be possible to confidently assign genes spanning a TAD boundary to one TAD or the other [48].

In order to assess the functional similarity between pairs of genes in TADs, where stated olfactory genes were removed from analysis. The olfactory genes have undergone a significant expansion in the mouse vs human genome. The human genome contains ~ 800 olfactory genes (of which < 400 are functional), whereas the mouse genome contains ~ 1400 olfactory genes (of which < 1050 are functional) [49]. Therefore, in order to make the findings of this study more relevant to human biology (where stated) the olfactory genes were masked. To achieve this MGI IDs (MGI v6.15) associated with Olfactory genes were downloaded by identifying any gene associated with the GO term: “olfactory receptor activity” [50–52] and IDs were converted to ensembl IDs using BioMart, ensembl IDs were used to remove genes from the analysis (1133 genes in total) [46].

### TAD randomisation and genome randomisation

We synthesised “random TADs” to serve as a null distributions to compare with TADs. To do this we generated two TAD randomisation strategies, each controlling for a different possible confounding signals. In the first strategy, which we refer to as “random TADs”, the position of each TAD was randomised within the same chromosome so that each TAD was randomly assigned to a new region of the same size as the original TAD. The new region was accepted only if it contained the same number of genes as the original TAD. TopDom TADs are non-overlapping, so overlapping was prohibited in random TopDom TADs. For each TAD, if a new region satisfying the criteria could not be found after 10,000 attempts, the TAD was excluded from the random TAD set. For TADs called using Arrowhead, we observed that the overlap structure was far more likely to be “nested” (one TAD falls completely within another) than “non-nested” (TADs overlap incompletely, with only part of the TAD falling within the bounds of the other) (Additional file 1: Fig. S1). To approximate this overlap structure in random TADs, every proposed new random TAD position was checked to see if it overlapped any existing random TADs. If it overlapped an existing random TAD in a “nested” fashion the overlap was always permitted. However, each random TAD was only permitted to overlap existing TADs in a “non-nested” fashion with 10% probability. Therefore, for most random TADs a new position was never accepted if it overlapped an existing TAD in a “non-nested” fashion thereby minimising this type of overlap. As with the random TopDom TADs, if a position fulfilling this criteria could not be found after 10,000 attempts, the TAD was excluded from the random TAD set. In the second randomisation method, which we refer to as “random genome TADs” the position of TADs was maintained along with the number of protein-coding genes within them, but the identity of the protein-coding genes on each chromosome was randomised. TAD randomisation methods were implemented using pybedtools [53].

TADs called using Arrowhead and TopDom were randomised 100 times each, generating 100 sets each of: random Arrowhead TADs, random TopDom TADs, Arrowhead random genome TADs and TopDom random genome TADs. Since, during the generation of each random TAD set, the algorithm randomises the position of each TAD in turn, the order of TADs was shuffled before the generation of every random TAD set. In order to test if 100 randomisation was enough, we plotted the median value of each measure investigated in this study with each added random TAD/random genome TAD set. For most measures the median begins to converge at fewer than 100 randomisations (Additional file 1: Fig. S10). The scripts required to run the randomisation method can be found at https://github.com/MRC-Harwell/TAD_randomisation.

To test how our TAD randomisation algorithm performs compared to other recently published methods we created TAD randomisation algorithms based on the descriptions in Nora et al. [17] and Rao et al. [4]. We used these algorithms to create example random TAD sets and compared them to an example random TAD set created using our method (Additional file 1: Fig. S1). In brief, in the Nora et al. [17] method, each TAD was randomised to a region on the same chromosome which contains the same number of genes and is the same length or smaller than the original TAD. We adapted this method to prevent overlapping when randomising TopDom TADs, if a non-overlapping TAD could not be placed after 10,000 attempts it was excluded from the TopDom random TAD set. In the Rao et al. [4] method, each TAD was randomised to a new position on the same chromosome but prevented from overlapping any gaps in the mm10 assembly (mm10 gaps were downloaded from the UCSC table browser [54]). This method was adapted to prevent overlapping when randomising TopDom TADs. Again, we set a cut off of 10,000 attempts to place each TAD before it was excluded from the TopDom random TAD set.

We compared the distance between pairs of genes in random TADs generated using our method to random TADs generated using the Nora et al. [17] method and the Rao et al. [4] method (Additional file 1: Fig. S1). Since the Nora el al. [17] method does not generate any TADs containing zero genes, TADs with zero genes were removed from all TAD sets. Regardless of the randomisation method used, we observed that the distance between genes in random TADs was always significantly different to the distance between genes in TADs. However, the effect size of these differences was smallest for random TADs produced by our method. This suggests that for this feature, random TADs produced by our method are the closest of the three methods to real TADs (Additional file 1: Fig. S1A).

We next compared the number of genes in random TADs. Again, TADs with zero genes were removed before comparison. Since in our randomisation method and in the Nora et al. [17] method, random TADs must contain the same number of genes as the original TADs, the only difference observed between real and random TADs produced by these methods were caused by TADs which could not be placed after 10,000 attempts and were therefore excluded. We observed no significant difference between the number of genes within Arrowhead TADs and random Arrowhead TADs using these methods. We also observed no significant difference between TopDom TADs and random TopDom TADs produced by our method. However, TopDom random TADs generated using the Nora et al. [17] method were significantly different from real TopDom TADs for both ESC (*p*-value < 0.05) and cortical neuron (*p*-value < 0.01). We also observed a significant difference (*p*-value < 0.001) between the number of genes in random TADs generated by the Rao et al. [4] method and TADs (Additional file 1: Fig. S1B). Therefore, random TADs produced using our method best reflect the number of genes in TADs.

The overlap structure of real Arrowhead TADs favours nested TADs. To assess how well each randomisation method approximates the overlap structure of Arrowhead TADs, we selected all TADs/random TADs which were involved in any type of overlap. We then annotated them according to whether they were involved in nesting overlaps, non-nesting overlaps or both. We found that our randomisation method best approximates the overlap structure of Arrowhead TADs. Random Arrowhead TADs generated using the Rao et al. [4] or the Nora et al. [17] method contain more non-nesting overlaps than Arrowhead TADs (Additional file 1: Fig. S1C).

### Pairs of genes with shared ancestry

The functional similarity of genes within a TAD was measured by assessing the similarity of every possible pair of genes within the same TAD. Gene pairs which have shared ancestry, i.e. paralogs, are expected to be very functionally similar. In order to assess whether genes within TADs are more functionally similar irrespective of shared ancestry, (where stated) paralogous gene pairs were removed from the analysis. To do this mouse paralogous gene pairs were downloaded from BioMart (Ensembl release 98) [46].

### Constraint score

Selective constraint was quantified as a nonsynonymous z-score across 15,648 mouse genes [33] by considering variation between 36 strains of mice commonly used for genetic research [55]. In brief, constraint was quantified for each gene as the deviation of the observed number of nonsynonymous variants relative to the expected number given no selection, which was determined by the average rate of synonymous fixation across all strains. Genes that have a greater relative depletion of nonsynonymous variants are considered more constrained by negative selection. Genes were defined by their Ensembl canonical transcripts and were filtered if less than 90% of their translated sequence has coverage greater than or equal to 10X in over 90% of the mouse strains. Ensembl transcript IDs were mapped to gene IDs (Ensembl release 99). When calculating the average constraint of genes sharing a TAD, if any genes mapped to multiple transcripts the constraint associated with all transcripts was considered.

### Functional enrichment analysis

Functional enrichment analysis was undertaken using the g:profiler r package (version e100_eg47_p14_021df73) [56] and Biological processes GO terms. The background was set to protein-coding genes and electronic annotations were removed. The plots include the (max) top 25 most significant GO terms passing a *p*-value threshold of < 0.05 (multiple testing corrected using the “gSCS” option). The similarity between each of the significant GO terms was calculated using the r package GOSemSim [57] (using the “Jiang” similarity method as the “measure” parameter and “NULL” as the “combine” parameter). These similarity scores were then used as input into hclust clustering, the order resulting from clustering was used to order the GO terms in the plots.

### Gene co-expression

FPKM counts from RNA-seq data generated alongside the Hi-C data were downloaded from GEO (GEO Accession Number: GSE96107). The data consisted of two replicates each for ESC, neural progenitor cells (NPC), and cortical neurons. Genes with an FPKM value < 1 were treated as having 0 expression. Gene co-expression was calculated across all 6 samples using Spearman’s rank correlation coefficient between all pairs of genes. Correlation coefficients calculated from this data indicate the similarity of expression over three cell types.

To guard against the possibility that TAD structure may differ between cell types we also downloaded RNA-seq data from the closest matching tissues/cell types to ESCs and cortical neurons which had at least 3 samples (required for the correlation analyses) from the Encode project [34] and GEO [36]. To assess the expression correlation within cortical neuron TADs we downloaded encode forebrain RNA-seq. The forebrain RNA-seq was generated with two replicates each, of embryos of varying ages. Accession numbers: ENCFF302TQO, ENCFF976OLT, ENCFF895JXR, ENCFF227HKF, ENCFF340XFQ, ENCFF484AOO, ENCFF601JPN, ENCFF413BXV, ENCFF465SNB, ENCFF567AFL, ENCFF590FAC, ENCFF745ZJF, ENCFF763GXJ, ENCFF804FTJ, ENCFF816CVP and ENCFF918QNL [34, 35]. Genes with an FPKM value < 1 were treated as having 0 expression. To assess the expression correlation within ESC TADs we downloaded mouse ESCs differentiating to PGC RNA-seq data. The ESCs differentiating to PGC RNA-seq data were generated with three replicates each of ESC, epiblast like cells (day 2), PGC (day 4) and PGC (day 6) [37]. Processed RNA-seq data were downloaded from GEO accession GSE86903, data were provided as log_2_(RPM) which we converted to RPKM for analysis. Genes with an RPKM value < 1 were treated as having 0 expression. The original GSE86903 data were provided with gene names rather than IDs, gene names were converted to ensembl IDs by matching the gene names and coordinates to the ensembl IDs from ensembl release 77 (the archive predating the data to provide more complete mapping).

### GO semantic similarity

The R package GOSemSim [57] was used to calculate GO semantic similarity scores. For each pair of genes the GO terms assigned to them were compared using the Jiang method. If genes were associated with multiple GO terms, scores were combined using “best match average”. Pairs of genes where one or both have no annotated GO terms were excluded from the analysis as no score could be generated. We first calculated the similarity score between all pairs of genes in the genome using each of the MF, Biological process (BP) or Cellular component (CC) ontologies. We then plotted these scores for all autosomal genes, autosomal genes minus olfactory genes, and autosomal genes minus olfactory genes and paralogous pairs, against genomic distance in the real genome compared to the median distance in 1000 random genomes. We found that, for scores calculated with BP and CC, once olfactory genes and paralogous gene pairs have been removed there is no association between GO similarity and distance. This suggests that similarity in these scores is driven by paralogous pairs and the olfactory genes. We therefore moved forward using only MF in our analysis (Additional file 1: Fig. S11).

### Shared pathways

Kegg pathways were downloaded from org.Mm.eg.db (v. 3.8.2) [58]. The Kegg pathway data are very sparse and many genes do not have a pathway annotation. In order to account for this, the amount of pairs of genes sharing at least one pathway annotation was considered as a proportion of all pairs of genes with at least one pathway annotation each.

### Shared protein–protein interactions (PPIs)

PPI data were downloaded for mm10 from string v11 [59]. Only interactions with the mode “binding” were selected so that only direct/physical interactions (rather than functional interactions which may not require physical contact) were included. Protein IDs were mapped to gene IDs using the BioMart r package (Ensembl v98). Similarly to pathways, PPI data are very sparse and many genes are unannotated. Therefore, as with pathways the amount of pairs of genes with a PPI was considered as a proportion of all pairs of genes with at least one PPI annotation each.

### Random permutation testing

For expression correlation, GO semantic similarity, proportion of gene pairs sharing a pathway and proportion of genes pairs sharing a PPI, we have plotted the functional score against binned genomic distance in the real genome and compared it to the median value of the functional score in 1000 random genomes. In these analyses for each bin, we have established whether there is a significant difference between the functional score in the real genome compared to the distribution of scores in 1000 random genomes using permutation testing. For each bin this has been calculated as follows: sum(values in the random genome ≥ value in the real genome)/1000. *P*-values were then FDR corrected.

### Effect size

Effect size, *r*, was calculated using the r package rcompanion. A positive effect size indicates that the value associated with TADs is greater (than random TADs/random genome TADs) whereas a negative effect size indicates that the value associated with TADs is lesser (than random TADs/random genome TADs). The larger the value the larger the effect size.

### Hi-C matrix visualisation

Hi-C matrix figures were made using Juicebox 1.11.08 [60].

## Supplementary Information

**Additional file 1.** Fig. S1–S12.

**Additional file 2.** Table S1–S7.

## Data Availability

Publicly available data was utilised in this manuscript as indicated by accession numbers in “Methods”.

## Abbreviations

CN: Cortical neurons
ESC: Embryonic stem cells
FPKM: Fragments Per Kilobase of transcript per Million mapped reads
GEO: Gene Expression Omnibus
GO: Gene Ontology
MAPQ: MAPping Quality
MF: Molecular function
NPC: Neural progenitor cells
NS: Not significant
PGC: Primordial germ cell
PPI: Protein–protein interactions
RPKM: Reads Per Kilobase of transcript per Million mapped reads
TAD: Topologically associating domains
