# Supplementary material for: Making sense of the linear genome, gene function and TADs
Source: Epigenetics Chromatin. 2022 Jan 29;15:4. doi: 10.1186/s13072-022-00436-9 (PMC8800309; doi:10.1186/s13072-022-00436-9)
Supplement: Supplementary file 1 — Additional file 1. Fig. S1–S12. [file 13072_2022_436_MOESM1_ESM.docx]

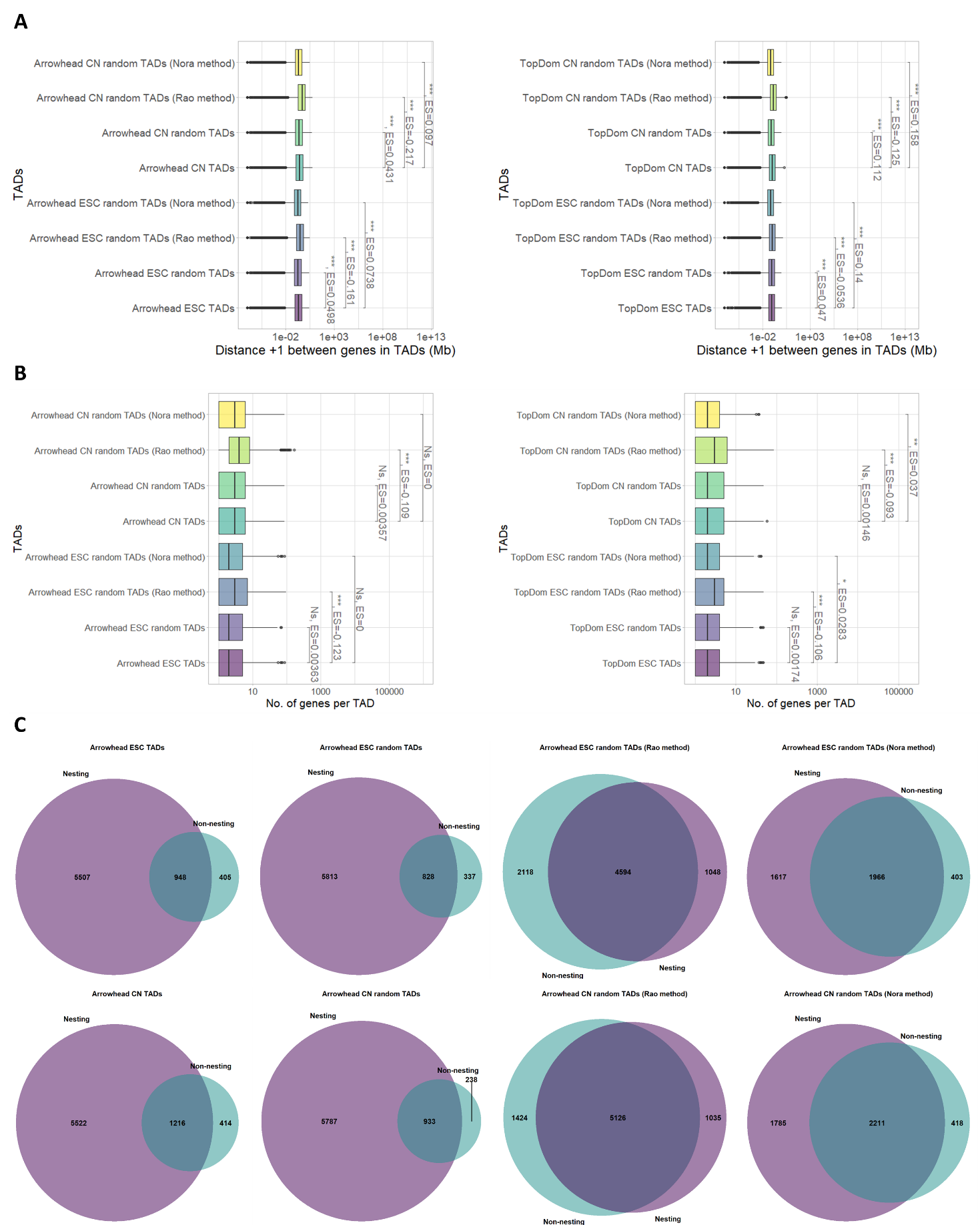


**Fig. S1. TAD randomisation method compared to recently published methods (autosomal TADs).** Comparison of Arrowhead or TopDom TADs, and an example set of random Arrowhead or TopDom TADs generated either: using our TAD randomisation method, as described in Nora et al. [17], or as described in Rao et al. [4]. A) Distance between pairs of genes in TADs/random TADs (log_10_ scale). TADs containing no genes were excluded (Wilcoxon test, p-value: p < 0.001 = ***, p < 0.01 = **, p < 0.05 = *, ES = Effect size calculated using r for Wilcoxon). B) Number of genes in TADs/random TADs. TADs containing no genes were excluded (Wilcoxon test, p-value: p < 0.001 = ***, p < 0.01 = **, p < 0.05 = *, NS = not significant, ES = Effect size calculated using r for Wilcoxon). C) Venn diagrams displaying the overlap structure of Arrowhead TADs and an example set of Arrowhead random TADs generated by each method. Non-Overlapping TADs were excluded. “Nesting” is defined as an overlap in which one TAD is contained entirely within another TAD, and “non-nesting” is defined as any incomplete overlap (i.e. only part of one TAD is contained within another TAD).


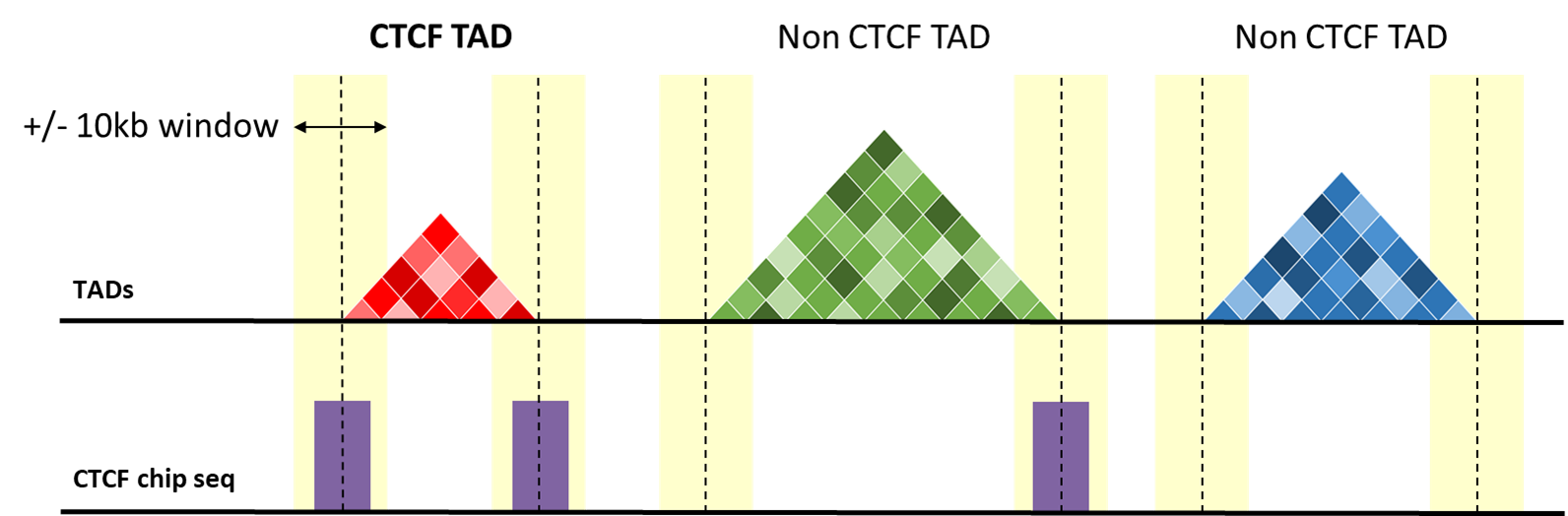


***Fig. S2. CTCF TAD selection. Schematic demonstrating how CTCF TADs are defined.*** *A TAD was annotated as a “CTCF TAD” if both boundaries fall within ± 10 kb of a CTCF peak in CTCF ChIP-seq data from the same cell type.*


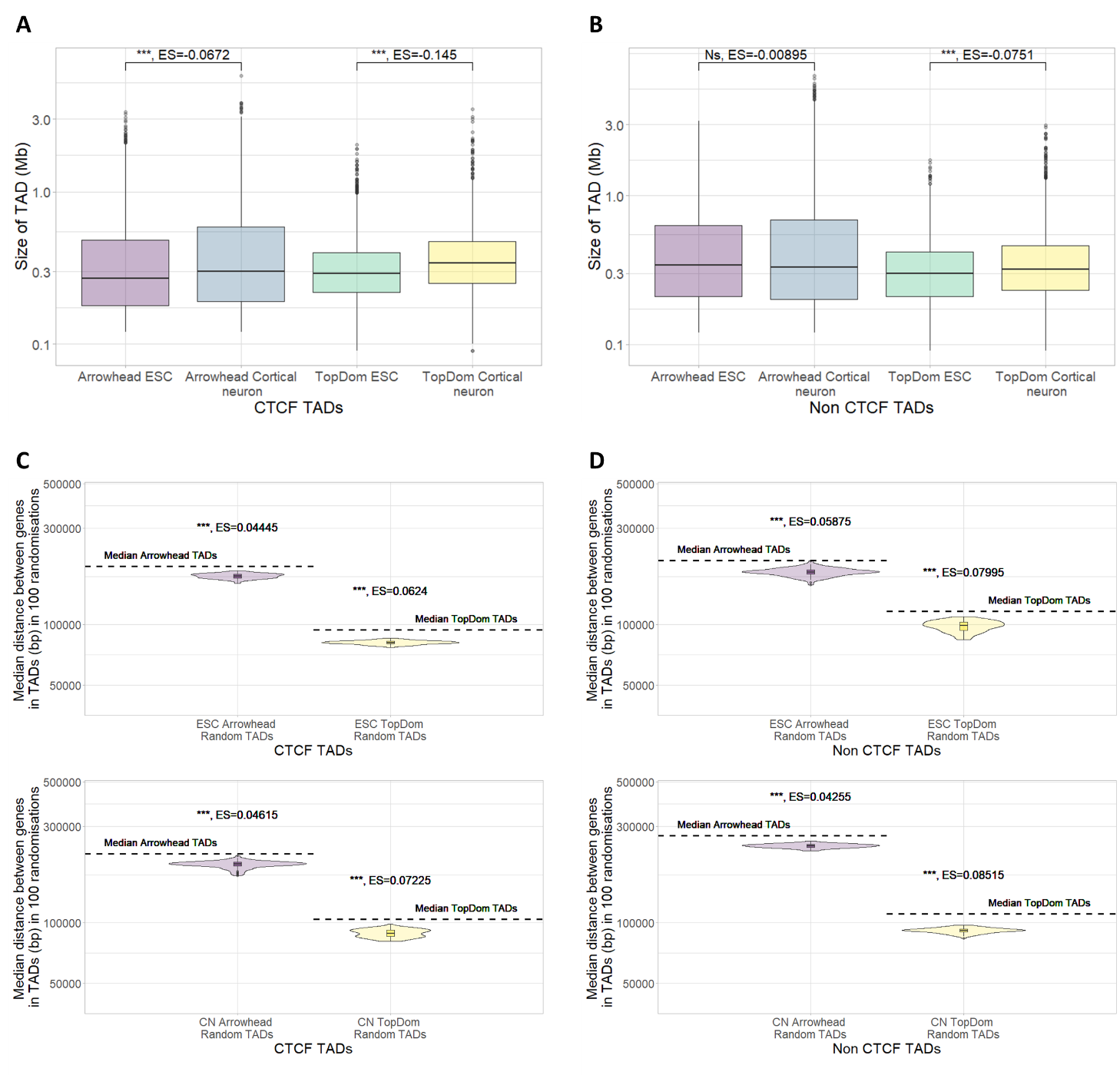


***Fig. S3. Features of autosomal TADs split into CTCF TADs and nonCTCF TADs.*** *A) Size of CTCF TADs called using Arrowhead and TopDom on ESC and cortical neuron Hi-C (plotted on a log_10_ scale) (Wilcoxon test, p-value: p < 0.001 = ***, p < 0.01 = **, p < 0.05 = *, ES = Effect size calculated using r for Wilcoxon). B) Size of nonCTCF TADs called using Arrowhead and TopDom on ESC and cortical neuron Hi-C (plotted on a log_10_ scale) (Wilcoxon test, P-value: p < 0.001 = ***, p < 0.01 = **, p < 0.05 = *, NS = not significant, ES = Effect size calculated using r for Wilcoxon). C) Median distance between gene start coordinates in CTCF TADs (dotted line) vs the median distance between genes in 100 sets of random TADs (plotted on a log_10_ scale). (Wilcoxon test, Median p-value: p < 0.001 = ***, p < 0.01 = **, p < 0.05 = *, ES = Median effect size calculated using r for Wilcoxon). D) Median distance between gene start coordinates in nonCTCF TADs (dotted line) vs the median distance between genes in 100 sets of random TADs (plotted on a log_10_ scale). (Wilcoxon test, Median p-value: p < 0.001 = ***, p < 0.01 = **, p < 0.05 = *, ES = Median effect size calculated using r for Wilcoxon).*


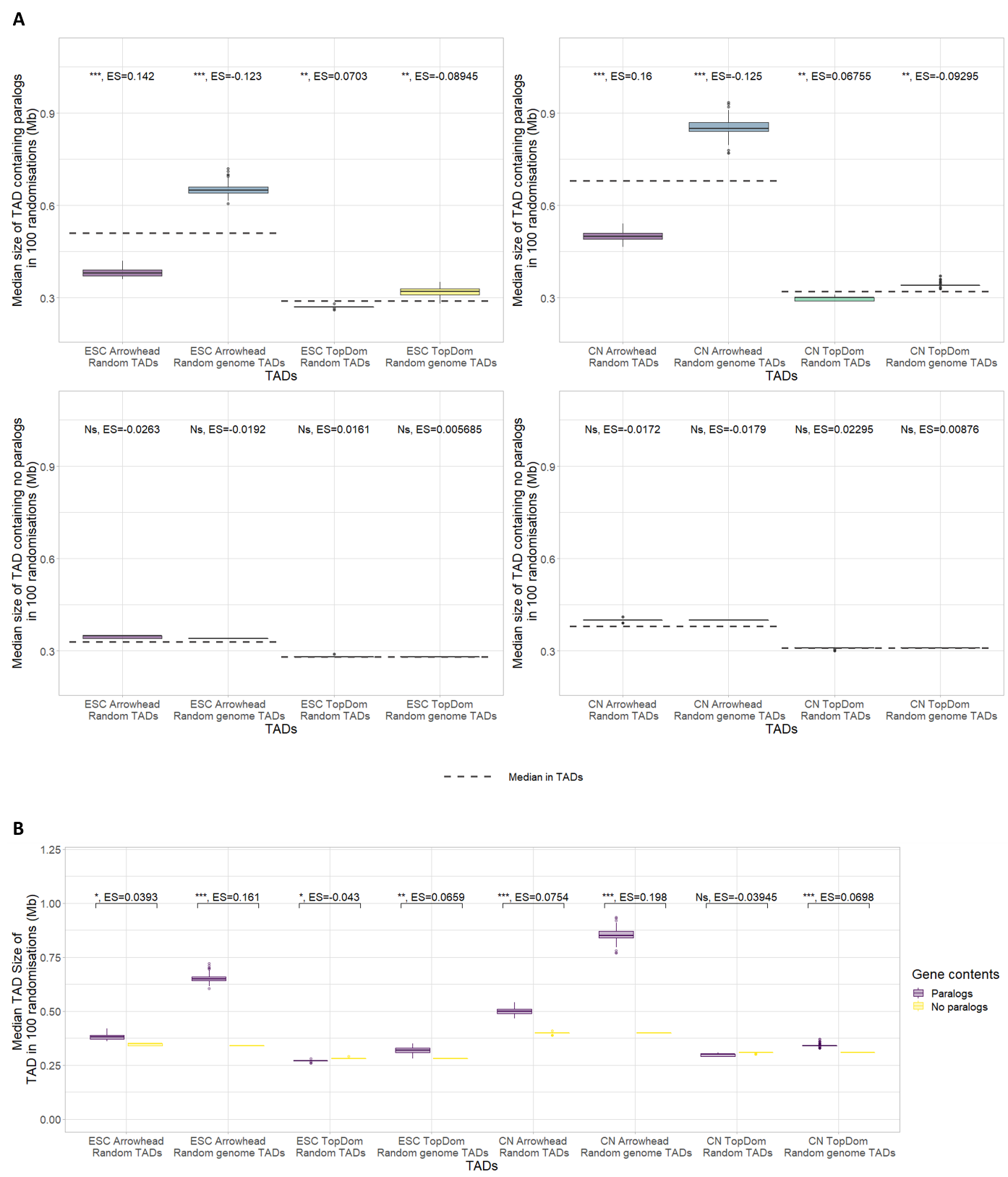


**Fig. S4. Size of autosomal random TADs and random genome TADs containing a pair of paralogs.** A) Median size of TADs (with > 1 gene) (dotted lines) vs the median size of TADs (with > 1 gene) in 100 sets of random TADs or random genome TADs, for TADs containing a pair of paralogs (top) or no paralog pairs (bottom) (Wilcoxon test, median p-value: p < 0.001 = ***, p < 0.01 = **, p < 0.05 = *, NS = not significant, ES = Median effect size calculated using r for Wilcoxon). B) Median size of random or random genome TADs containing pairs of paralogs vs containing no pairs of paralogs (with > 1 gene) in 100 sets of random TADs and random genome TADs (Wilcoxon test, median p-value: p < 0.001 = ***, p < 0.01 = **, p < 0.05 = *, NS = not significant, ES = median effect size calculated using r for Wilcoxon).


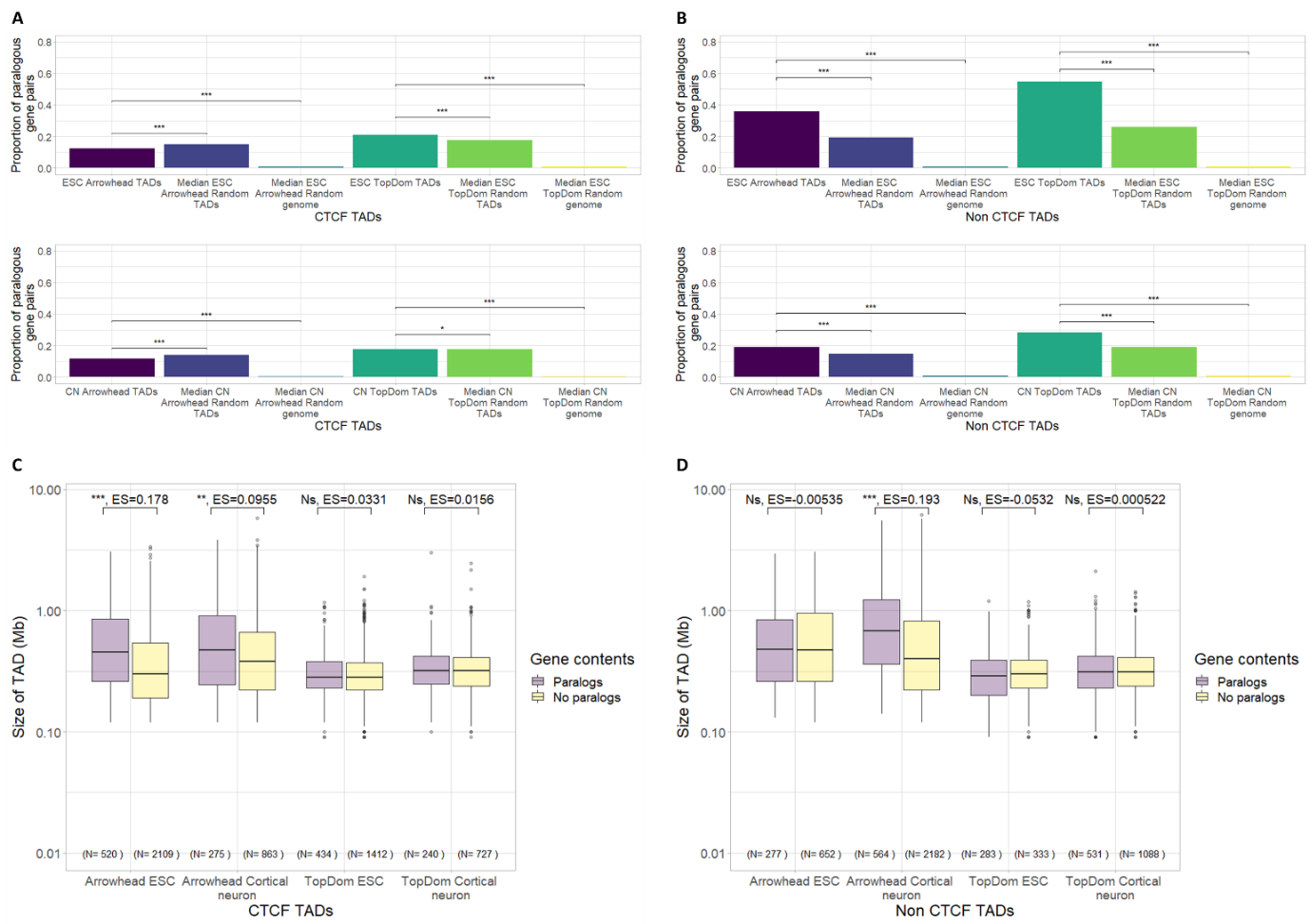


**Fig. S5. Paralogous gene pairs in autosomal CTCF and nonCTCF TADs.** A) Proportion of paralogous gene pairs in CTCF TADs or B) nonCTCF TADs compared to the median proportion in 100 sets of random TADs, and the median proportion in 100 sets of random genome TADs in ESCs or cortical neurons (CN) (Fisher’s exact test, Median p-value: p < 0.001 = ***, p < 0.01 = **, p < 0.05 = *). C) Size of CTCF TADs or D) nonCTCF TADs containing pairs of paralogs vs (with > 1 gene) containing no pairs of paralogs (plotted on a log_10_ scale) (Wilcoxon test, p-value: p < 0.001 = ***, p < 0.01 = **, p < 0.05 = *, NS = not significant, ES = Effect size calculated using r for Wilcoxon).


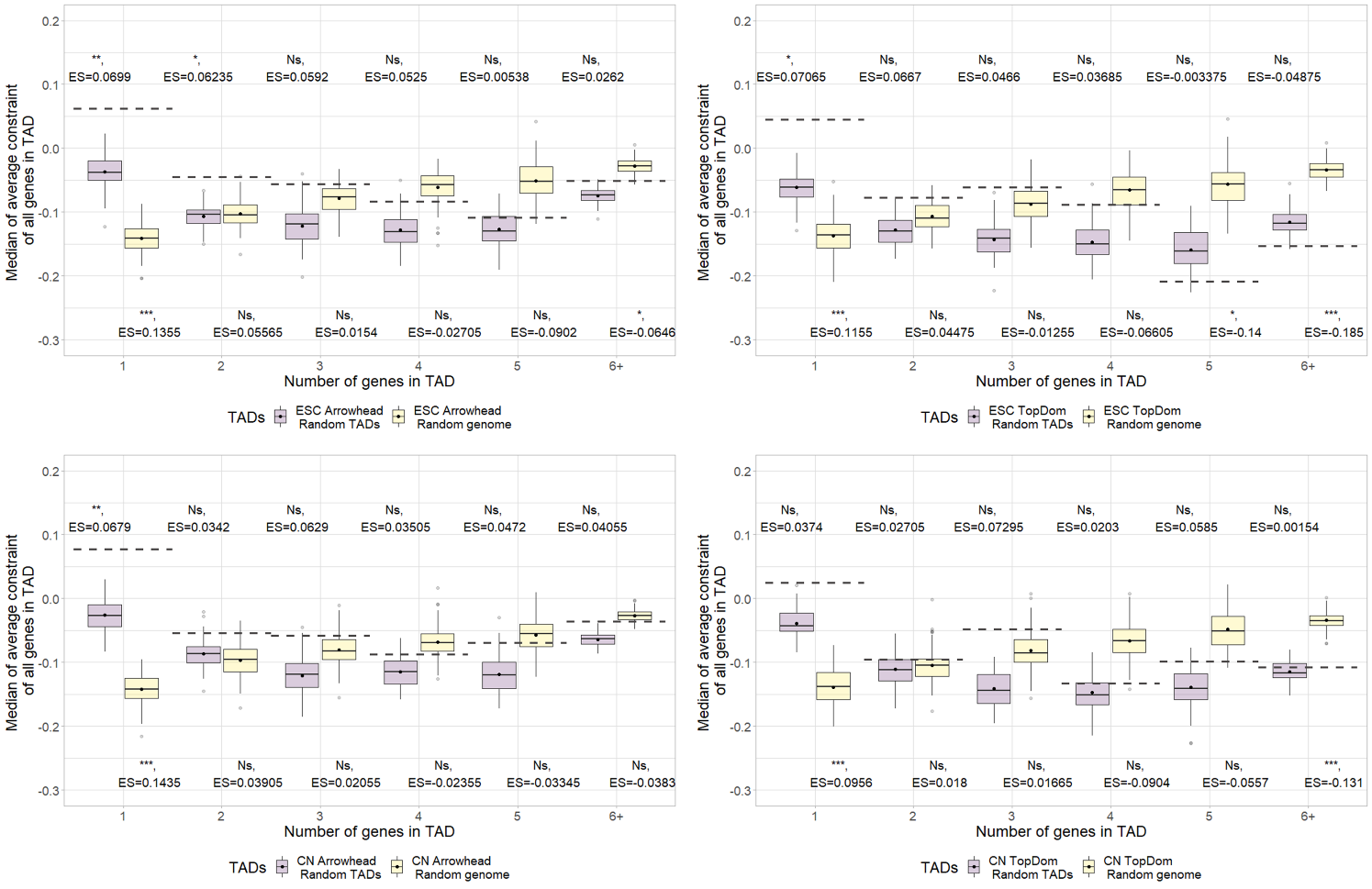


**Fig. S6. Average constraint of genes in TADs vs random TADs/random genome autosomal TADs.** The average gene constraint score for all genes within a TAD/random TAD was calculated to create a score per TAD. TADs were binned based on the total number of genes they contained and the median score for each bin was plotted. The dotted line shows the median score in TADs and the distributions show the median scores in 100 sets each of: random TADs and random genome TADs. Dots indicate mean of the distribution (Wilcoxon test, FDR corrected median p-value (corrected for 6 tests, corresponding to the number of groups on the x axis): p < 0.001 = ***, p < 0.01 = **, p < 0.05 = *, NS = not significant, Median ES = Effect size calculated using r for Wilcoxon). P-values shown above the bars for random TADs and below the bars for random genome TADs.


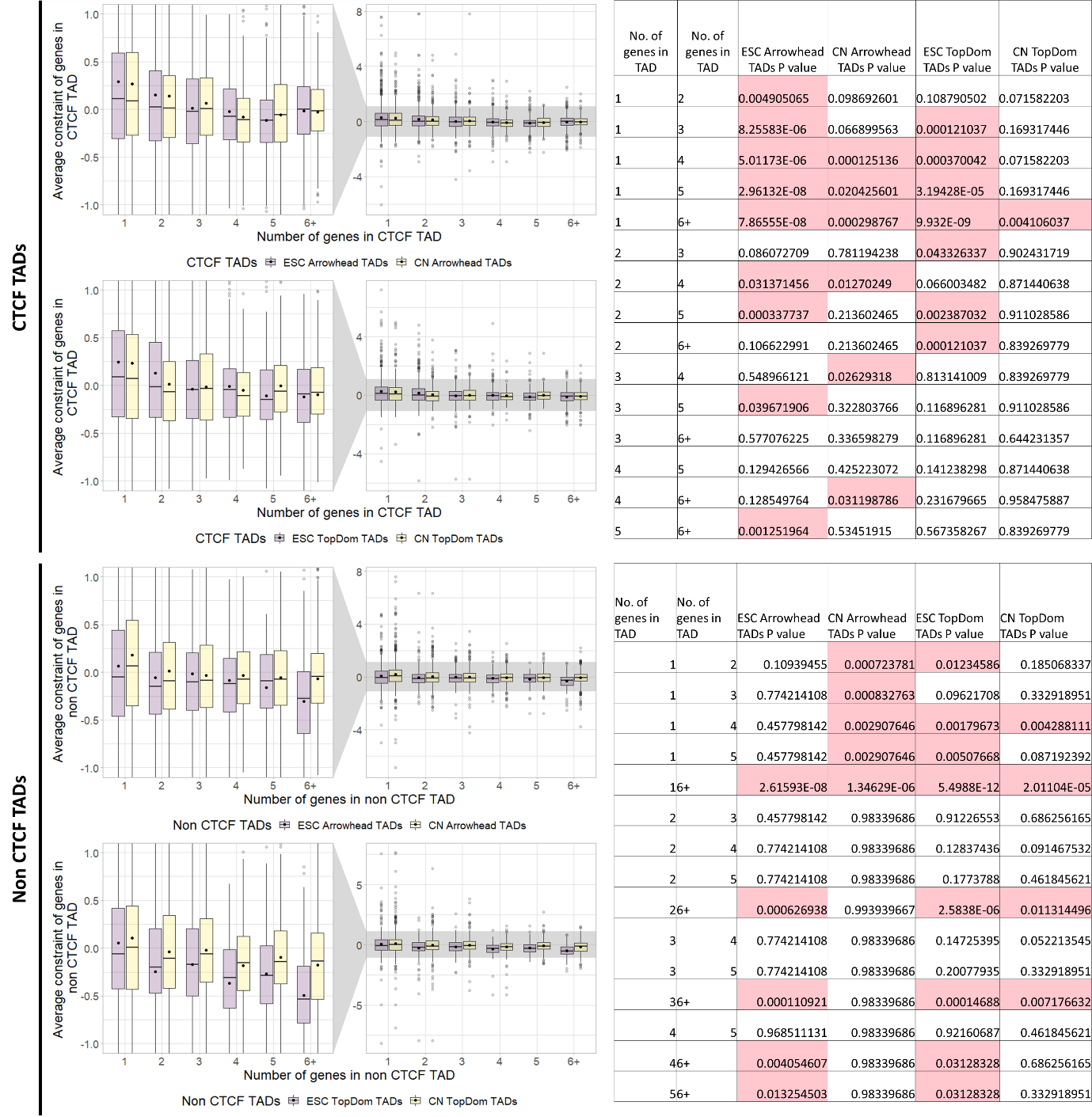


**Fig. S7. Average constraint in CTCF autosomal TADs or nonCTCF autosomal TADs binned by number of genes.** Distribution of mean constraint scores of all genes in CTCF TADs (top) or nonCTCF TADs (bottom). Dots indicate the mean of the distribution. Tables showing FDR corrected p-values of differences between the groups in the graphs calculated with the Wilcoxon test. Significant p-values are highlighted red.


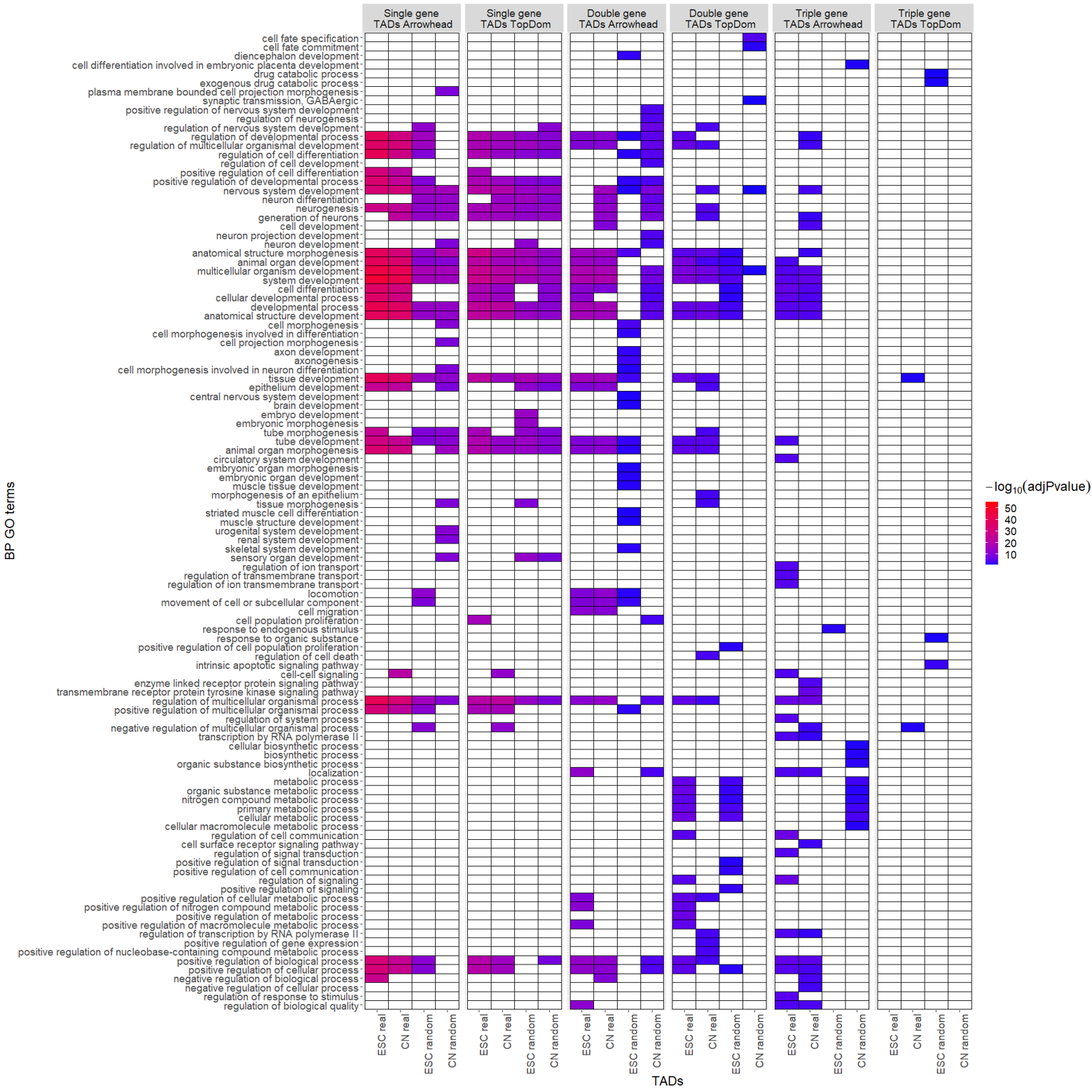


**Fig. S8. Biological processes GO term functional enrichment of genes in Arrowhead and TopDom TADs and Random TADs.** GO term enrichments calculated for genes within Arrowhead TADs, TopDom TADs, one example of random Arrowhead TADs and one example of random TopDom TADs containing a single gene, two genes, or three genes. Only the top 25 most significant (p < 0.05 multiple testing corrected using the “gSCS” option) GO terms are shown for each gene set.


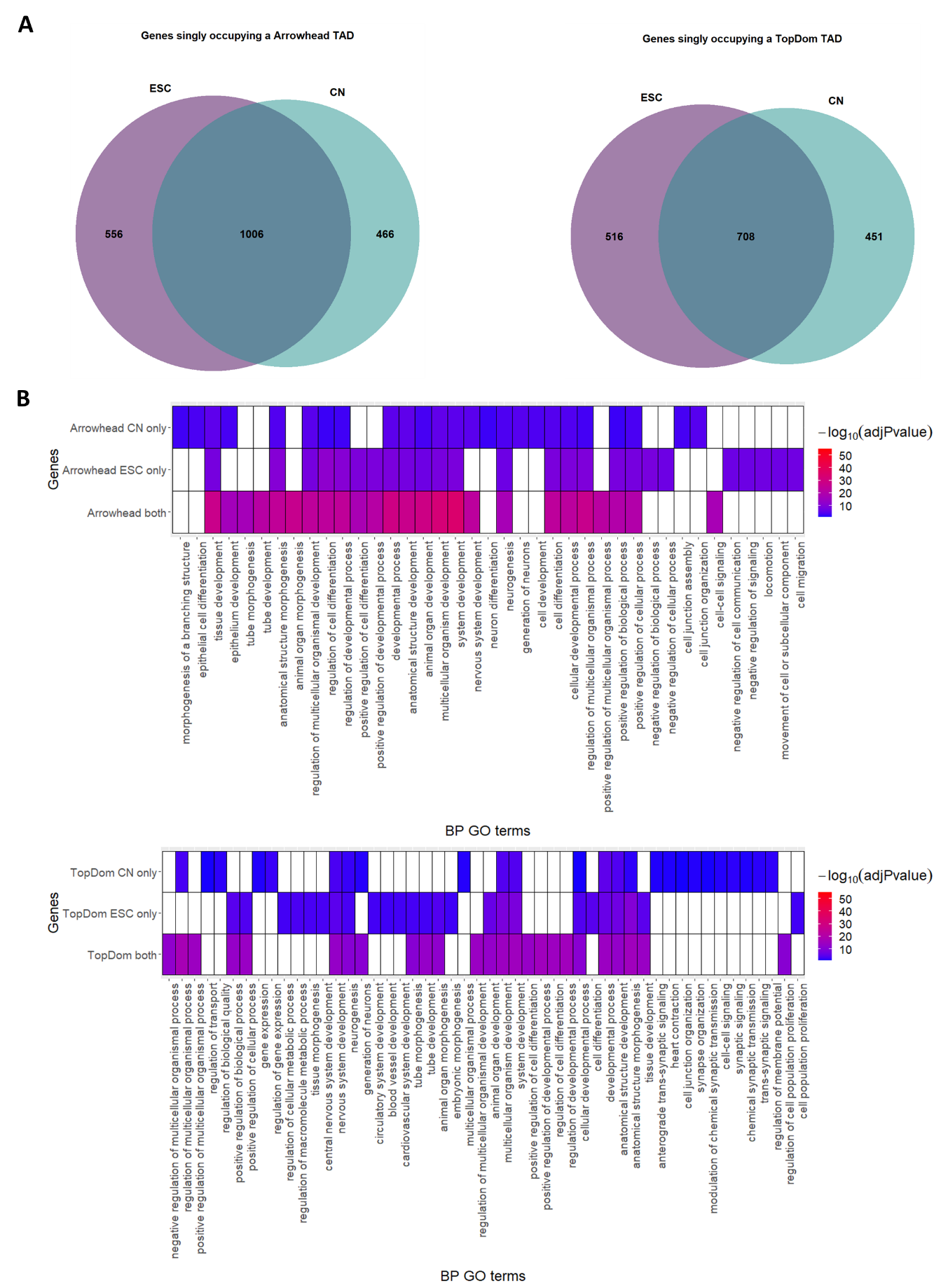


**Fig. S9. Comparison of genes singly occupying a TAD in ESC and cortical neuron.** A) Venn diagrams comparing genes singly occupying a TAD between ESC and cortical neuron for Arrowhead TADs (left) and TopDom TADs (right). B) GO term functional enrichment of genes singly occupying a TAD in cortical neuron only, ESC only or both cortical neuron and ESC for Arrowhead (top) and TopDom (bottom). Only the top 25 most significant (p < 0.05 multiple testing corrected using the “gSCS” option) GO terms are shown for each gene set.


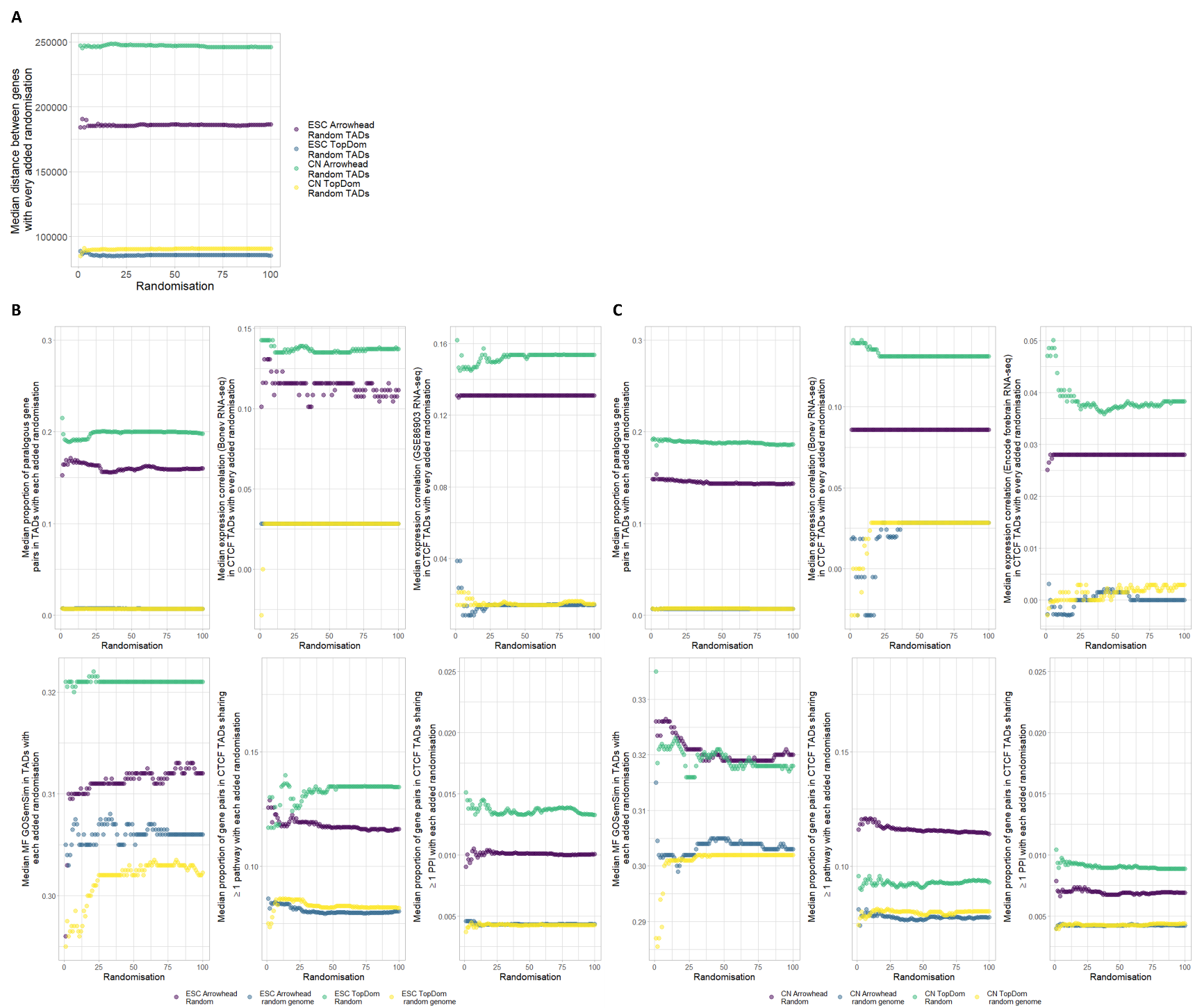


**Fig. S10. Median value of measures tested, in random TADs with each added random TAD set: For each measure the median begins to converge before 100 randomisations.** Measures: A) median distance between genes B) For ESC random TADs/random genome TADs: median proportion of paralogous gene pairs, median expression correlation between gene pairs calculated using RNA-seq from Bonev et at. or GSE86903 et al., median MF GO semantic similarity between gene pairs, median proportion of pairs of genes sharing ≥ 1 pathway and median proportion of pairs of genes sharing ≥ 1 PPI. C) For cortical neuron random TADs/random genome TADs: median proportion of paralogous gene pairs, median expression correlation between gene pairs calculated using RNA-seq from Bonev et at. or encode forebrain, median MF GO semantic similarity between gene pairs, median proportion of pairs of genes sharing ≥ 1 pathway and median proportion of pairs of genes sharing ≥ 1 PPI.


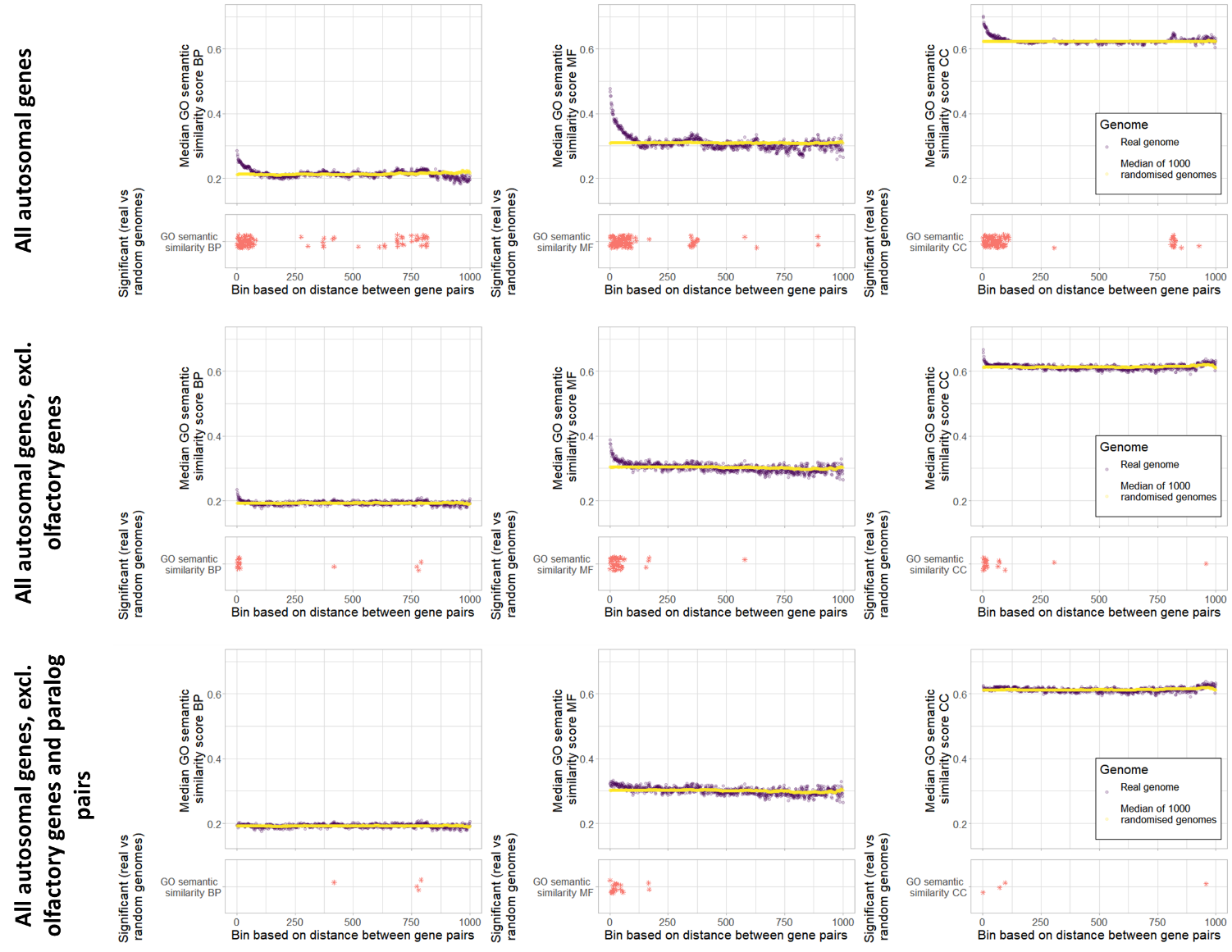


**Fig. S11. Pairwise GO similarity scores against genomic binned distance.** Distribution of GO semantic similarity for pairs of genes binned by distance in the real genome vs 1000 random genomes. In the top row of panels all autosomal genes are included, in the middle row olfactory genes are excluded and in the bottom row all olfactory genes are excluded along with any pairs of paralogs. Significance/bottoms panels: Stars indicate bins with a significantly higher GO semantic similarity in the real genome vs 1000 random genomes (FDR corrected p-value < 0.05).


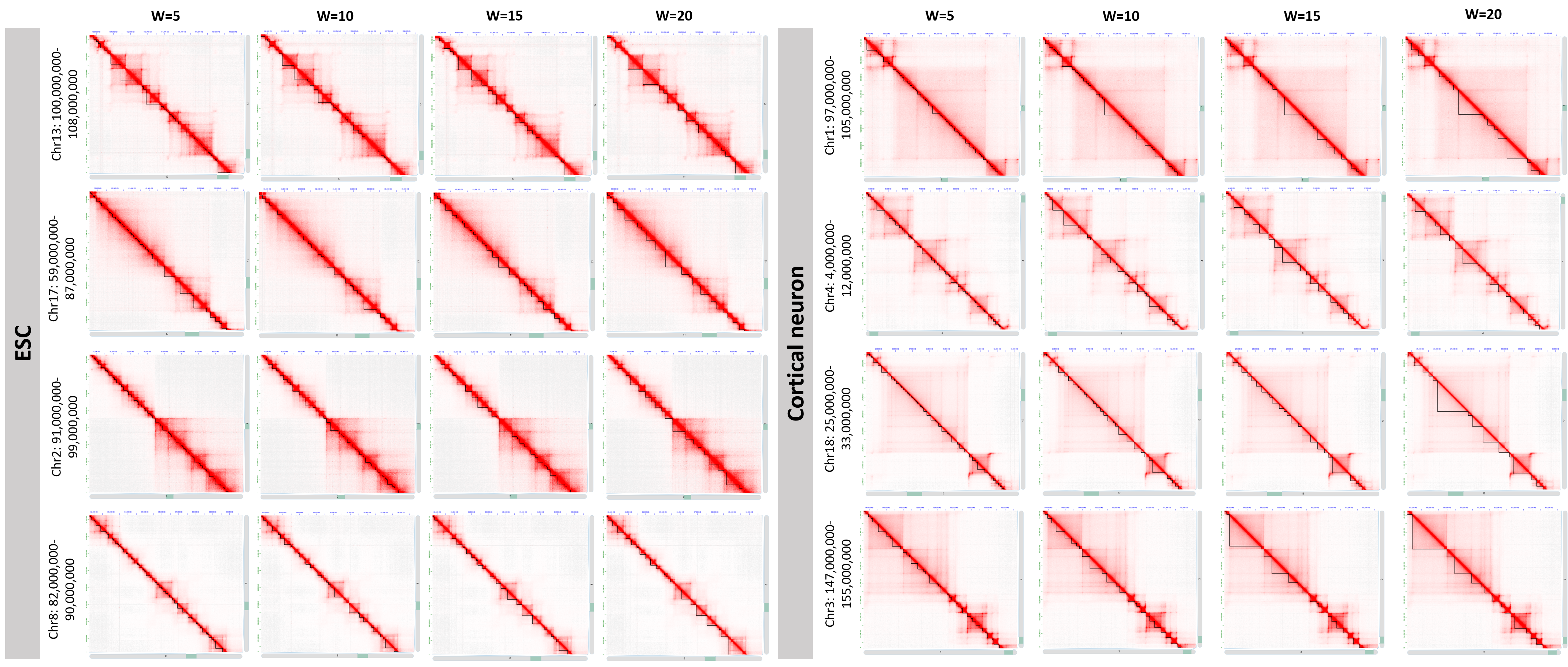


***Fig. S12. TopDom TADs called with w = 5, 10, 15, 20.*** *TopDom TADs in four regions in ESC (left) and four regions in cortical neuron (right).*
